# Tamil Nadu Pregnancy and Heart Disease Registry (TNPHDR): design and methodology

**DOI:** 10.1186/s12884-021-04305-3

**Published:** 2022-01-29

**Authors:** Justin Paul Gnanaraj, S Anne Princy, Karen Sliwa-Hahnle, Sowmya Sathyendra, Nambirajan Jeyabalan, Ragothaman Sethumadhavan, Selvarani G, N. Sumathi, Vinotha S, Pachaiappan P, Vimali Murali, Shanthirani B, Gomathi T, Muthuprabha P, Panniyammakal Jeemon, E. Elavarasi, Rajarajeshwari R, Vijaya S, Kanmani K, the TNPHDR investigators

**Affiliations:** 1 Madras Medical College and Research Institute, Chennai, India; 2 Tamil Nadu Government Multi Super Speciality Hospital, Chennai, India; 3 Hatter Institute for Cardiovascular Research in Africa, Cape Town, South Africa; 4 Christian Medical College, Vellore, India; 5 Coimbatore Medical College, Coimbatore, India; 6 Government Medical College, Chengalpattu, India; 7 Government Medical College, Madurai, India; 8 Government KAPV Medical College, Tiruchirappalli, India; 9 Government MKM Medical College, Salem, India; 10 Government Medical College, Theni, India; 11 Government Medical College, Tanjore, India; 12 Tirunelveli Medical College, Tirunelveli, India; 13 Sree Chitra Tirunal Institute for Medical Sciences and Technology, Trivandrum, India

**Keywords:** Pregnancy, Heart disease, Maternal outcome, Mortality, Risk prediction

## Abstract

**Background:**

Cardiac disease in pregnancy is a major contributor to maternal mortality in high, middle and low-income countries. Availability of data on outcomes of pregnancy in women with heart disease is important for planning resources to reduce maternal mortality. Prospective data on outcomes and risk predictors of mortality in pregnant women with heart disease (PWWHD) from low- and middle-income countries are scarce.

**Methods:**

The Tamil Nadu Pregnancy and Heart Disease Registry (TNPHDR) is a prospective, multicentric and multidisciplinary registry of PWWHD from 29 participating sites including both public and private sectors, across the state of Tamil Nadu in India. The TNPHDR is aimed to provide data on incidence of maternal and fetal outcomes, adverse outcome predictors, applicability of the modified World Health Organization (mWHO) classification of maternal cardiovascular risk and the International risk scoring systems (ZAHARA and CARPREG I & II) in Indian population and identify possible gaps in the existing management of PWWHD. Pregnancy and heart teams will be formed in all participating sites. Baseline demographic, clinical, laboratory and imaging parameters, data on counselling received, antenatal triage and management, peripartum management and postpartum care will be collected from 2500 eligible participants as part of the TNPHDR. Participants will be followed up at one, three and six-months after delivery/termination of pregnancy to document study outcomes. Predictors of maternal and foetal outcome will be identified.

**Discussion:**

The TNPHDR will be the first representative registry from low- and middle-income countries aimed at providing crucial information on pregnancy outcomes and risk predictors in PWWHD. The results of TNPHDR could help to formulate steps for improved care and to generate a customised and practical guideline for managing pregnancy in women with heart disease in limited resource settings.

**Trial registration:**

The TNPHDR is registered under Clinical Trials Registry-India (CTRI/2020/01/022736).

**Supplementary Information:**

The online version contains supplementary material available at 10.1186/s12884-021-04305-3.

## Background

Cardiac disease in pregnancy presents a great challenge, since it involves a complex interplay of both obstetric and cardiovascular management of the mother and the fetus. Pregnancy imposes significant physiologic and hemodynamic changes on the cardiovascular system in normal women. Women with heart disease (WWHD) may not be able to cope up with the greater demands imposed on the cardiovascular system by these physiological changes during pregnancy. The incidence of pregnancy complicated by heart disease is on the rise due to multiple factors including increased prevalence of adverse lifestyle risk factors, delayed marriage and conception, increased awareness and early diagnosis of genetic conditions and improved survival of women with congenital heart diseases [1, 2].

Recent data show that mortality in WWHD during pregnancy is increasing, both in high and low-income countries [3]*.* Cardiac disease is one of the commonest cause of maternal deaths in high-income countries such as the United Kingdom, the United States of America and the Nordic countries [4–6]. With obstetric transition taking place related to improved management of bleeding, infective and hypertensive causes of maternal death, cardiovascular disease is becoming an increasingly important non-obstetric cause of maternal death in low-and middle-income countries (LMICs) [7]. Additionally*,* pregnant women with heart disease (PWWHD) from LMICs often do not receive optimal pre-conceptional counselling and care [8] . Further, diagnosis of heart disease for the first time during pregnancy and lack of availability / affordability to appropriate care [9–11] contribute to adverse outcomes in pregnancy.

To continue improvement in line with millennium development goals by the World Health Organization (WHO) in reducing global maternal mortality ratio (MMR) [12], each country/state/health care region has to plan its customised steps according to its current position in the obstetric transition, the prevailing health demographics, socio-cultural environment and the available health care infrastructure [7].

India is a nation with 28 states, 8 union territories, 22 official regional languages, [13] and significant political, geographical, cultural and educational diversity, which extends to its health care indicators like MMR and infant mortality rate. Some Indian states are still in stage III of obstetric transition with a MMR as high as 215 per 100,000 live births, while few others have already moved into stage IV [7] with a MMR as low as 43/100,000 live births [14]. States with lower MMR have to shift focus to target reduction of deaths due to heart diseases and other indirect causes, which needs a different set of interventions with multidisciplinary approach [15].

The state of Tamil Nadu in India is moving ahead in the obstetric transition with its MMR dropping from 111 in 2013 to 63 in 2017 [16] to the present level of 57 per 1,00,000 live births in 2019–2020. The contribution from heart disease to maternal mortality, which doubled over the last ten years from 4.56% to 10.36%, is likely to approach that of high-income countries (15–25%) as the state of Tamil Nadu moves into stage IV of obstetric transition. Early identification of heart disease in pregnant women and ensuring appropriate management strategies may improve foetal and maternal outcomes. A recent analysis of maternal deaths from Nordic countries, which have the lowest MMR in the world, suggested that improvements in care could have made a difference in outcomes in at least 32% of maternal deaths due to heart disease [4]. Lack of multidisciplinary care, customised local guidelines, non-uniform distribution of health care facilities and poor awareness (among public, health care providers and policy makers) are the predominant reasons for higher mortality in pregnant women with heart diseases from LMICs. A significant number of maternal deaths due to heart disease could be prevented in LMICs by targeted steps to deliver improved health care. The latest guideline from European Society of Cardiology (ESC) on managing pregnant women with heart disease recognises the potential of surveys and registries in generating answers to crucial questions in management of pregnant women with heart diseases [17]. The Tamil Nadu Pregnancy and Heart Disease Registry (TNPHDR) was initiated to generate essential data for informed and data driven policy changes, introduce appropriately targeted steps for strengthening the healthcare infrastructure, improve capacity for appropriate training of the health care workers, and develop a new customised local guidelines for management of PWWHD. It may facilitate to reduce the contribution of heart diseases to maternal mortality and thus enhance the pace of the obstetric transition to stage IV in this region.

## Methods/Design

The TNPHDR is aimed to (i) analyze the socio-demographic and clinical profile of various heart diseases observed during pregnancy, (ii) measure the fetal and maternal outcomes and identify predictors of those outcomes, (iii) examine the applicability of the modified World Health Organization (mWHO) classification of maternal cardiovascular risk [18, 19] (Table 1), and the appropriateness of the available risk prediction scoring systems based on data from high-income countries such as CARPREG I & II score [20, 21] and ZAHARA score [22] (Table 2) in LMICs, (iv) propose a risk scoring system unique for LMIC population, and (v) develop an evidence-based guideline for management of PWWHD in LMIC settings.

**Table 1:** Modified WHO Classification of heart disease in pregnancy [17, 18]

mWHO I	mWHO II	mWHO II-III
• Small or mild (Pulmonary stenosis, patent ductus arteriosus, mitral valve prolapse • Successfully repaired simple lesions (atrial or ventricular septal defect, patent ductus arteriosus, anomalous pulmonary venous drainage) • Atrial or ventricular ectopic beats-isolated	• Unoperated atrial septal defect or ventricular septal defect • Repaired tetralogy of Fallot • Most arrhythmias (supraventricular arrhythmias) • Turner syndrome without aortic dilatation	• Mild LV impairment (EF >45%) • Hypertrophic cardiomyopathy • Native or tissue valve disease not considered WHO I or IV (mild MS, moderate AS) • Marfan or other HTAD syndrome without aortic dilatation • Aorta <45 mm in BAV pathology • Repaired coarctation • Atrioventricular septal defect

**mWHO III**	**mWHO IV**
• Moderate LV impairment (EF 30–45%) • Previous PPCM without any residual LV impairment • Mechanical valve • Systemic RV with good or mildly decreased ventricular function • Fontan circulation- uncomplicated, stable patient • Unrepaired CHD • Other complex heart disease • Moderate MS • Severe asymptomatic AS • Moderate aortic dilatation (40–45 mm in Marfan syndrome or other HTAD; 45–50 mm in BAV, Turner syndrome ASI 20–25 mm/m^2^, TOF <50 mm) • Ventricular tachycardia	• Pulmonary arterial hypertension • Severe systemic ventricular dysfunction (EF <30% or NYHA class III–IV) • Previous PPCM with any residual LV impairment • Severe MS • Severe symptomatic aortic stenosis • Systemic RV with moderate or severely decreased ventricular function • Severe aortic dilatation (>45 mm in Marfan syndrome or other HTAD, >50 mm in BAV, Turner syndrome ASI >25 mm/m^2^, TOF >50 mm) • Vascular EDS • Severe (re)coarctation, Fontan with any complication

*PPCM* Peripartum Cardiomyopathy, *LV* Left Ventricle, *RV* Right Ventricle, *EF* Ejection fraction, *EDS* Ehlers Danlos syndrome, *MS* Mitral Stenosis, *AS *Aortic Stenosis, *HTAD* Heritable Thoracic Aortic Disease, *ASI *Aortic Size Index, *CHD* Cyanotic Heart Disease, *BAV* Bicuspid Aortic Valve

**Table 2:** Details of risk scores analysed in the TNPHDR (Parameters with points assigned)

CARPREG I Score [19]		CARPREG II Score [20]		ZAHARA Score [21]	
Prior cardiac events before pregnancy (HF, Stroke, TIA, or Arrhythmia)	− 1	Prior cardiac events or arrhythmia	− 3	Mechanical heart valve	− 4.5
Baseline NYHA > 2 or cyanosis SPO2 < 90%	− 1	Baseline NYHA III-IV or cyanosis	− 3	Left heart obstruction^**$**^	− 2.5
Left heart obstruction (MVA < 2 cm2, AVA < 1.5 cm2, LVOT pressure gradient > 30 mm of Hg by echo)	− 1	Mechanical heart valve	− 3	Prior Cardiac arrhythmia	− 1.5
Reduced LV ejection fraction < 40%	− 1	Systemic LV dysfunction*	− 2	Cardiac medication use before pregnancy	− 1.5
		High risk valve disease ^**#**^	− 2	Cyanotic CHD	− 1
		Pulmonary hypertension (RVSP > 49 mm of Hg)	− 2	NYHA ≥ 2	− 0.75
		High risk Aortopathy	− 2	Systemic AV Valve Regurgitation	− 0.75
		Coronary Artery Disease	− 2	Pulmonary AV Valve Regurgitation	− 0.75
		No prior Cardiac Intervention	− 1		
		Late Pregnancy Assessment	− 1		

* LV ejection fraction < 55%; ^**#**^ Aortic Valve area < 1.5 cm^2^ or subaortic gradient > 30 mm of Hg or Mitral stenosis < 2.0 cm^2^ or moderate to severe mitral regurgitation; ^**$**^Pressure Gradient > 50 mm of Hg or AVA < 1 cm^2^

*HF* Heart Failure, *TIA* Transient Ischemic Attack, *AVA* Aortic valve area, *LV* Left ventricle, *RVSP* right ventricular systolic pressure, *AV* Atrioventricular valve, *CHD* Congenital Heart disease, *MVA* Mitral Valve Area, *LVOT* Left Ventricular Outflow Tract

### Study settings

**Fig. 1:**
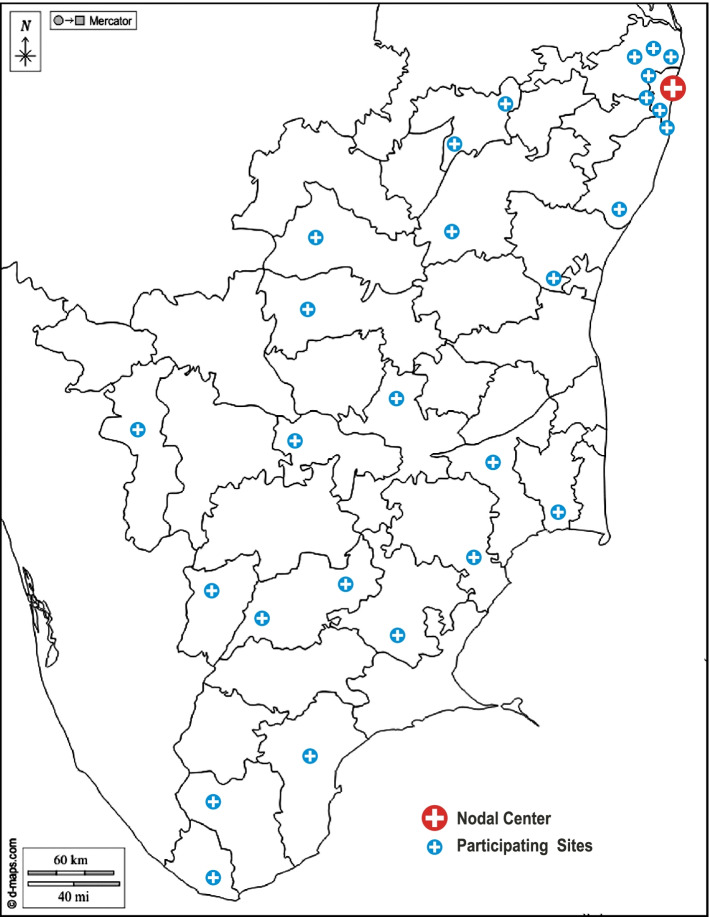
Map of participating sites in the TNPHDR. This figure shows the location of the 28 participating sites and the nodal center in the political map of Tamil Nadu, the southernmost state of India. ( adapted from www.d-maps.com)

All public and private hospitals with inhouse cardiology and obstetric services across the state of Tamil Nadu were eligible for enrollment as a participating site in the TNPHDR. The interested sites were requested to form a pregnancy and heart team with members from the departments of obstetrics, cardiology, pediatrics/neonatology and anesthesia to be responsible for the care of those participants enrolled in TNPHDR. The government of Tamil Nadu organized pregnancy and heart teams in all the eligible 23 government medical college teaching hospitals in Tamil Nadu and ensured their participation in the registry. Additionally, six leading private hospitals in Tamil Nadu also enrolled in the registry (Additional file 1, online supplement). The spread of the selected sites across the state of Tamil Nadu is given in Fig. 1. A premier public hospital at the state capital was chosen as the nodal coordinating center of TNPHDR. The pregnancy and heart team sensitization and TNPHDR sensitization training are conducted at each of the participating sites by the nodal center. Site investigators and co-investigators are chosen from the pregnancy and heart team of the participating sites.

### Enrollment, inclusion, and exclusion criteria

The TNPHDR is a prospective observational registry enrolling all antenatal women seeking outpatient or inpatient care in any of the participating sites with known or newly diagnosed structural heart disease, cardiac rhythm disorders or aortopathy/vascular diseases from 15^th^ of January 2020 to 31^st^ March 2021 or till at least 2500 eligible participants are enrolled. Antenatal patients will be enrolled at any trimester when they first enter the institution. Women with eligible heart disease seen after delivery will be included in the first six weeks postpartum. Inclusion for women with peripartum cardiomyopathy will be allowed up to six months postpartum. Collected data will be protected for privacy with all standard precautions. For example, each enrolled participant will be assigned a unique autogenerated TNPHDR identification number by which they will be identified subsequently. The unique number will remain the same despite inter-institutional transfer. Patients with pregnancy related complications like gestational hypertension, anemia, eclampsia, and gestational diabetes without structural heart disease will be excluded from the TNPHDR. Patients with trivial / mild regurgitation of the cardiac valves will be excluded, unless associated with other eligible heart diseases.

### Study tools

The “case report form (CRF) booklet” (provided as Additional file 2, online supplement) was developed based on the inputs of investigators of the participating sites and was finalized at the first investigators meeting organized at the National Health Mission, Government of Tamil Nadu, Chennai. The study variables will include baseline demographics, presenting symptoms, NYHA class, modified WHO classification, TNPHDR classification, clinical findings, baseline investigations, ECG and echocardiographic data, risk scores such as CARPREG I and CARPREG II and ZAHARA (Table 2). Data will be collected in the printed CRF booklets distributed to all participating sites. It will then be converted to an electronic format by using an online CRF web application platform. In addition, an Android/ iOS application will be also made available with facilities for both online and offline data entry. All the investigators will be provided with a username and password for restricted access to the data application. The nodal center will access, guide and support the online CRF data entry by the participating sites. The TNPHDR data will be stored in an encrypted format. The necessary backup of the database will be done every day in the evening (5 PM) to local servers at the nodal center as an added security measure. A standard operating procedure (SOP) will be formulated and circulated to all the participating centers (Additional file 3, online supplement) to enable understanding of the registry process and CRF entry.

### Antenatal triage of cardiac patients

The antenatal patients with cardiac disease will be initially triaged as high-risk cardiac illness and low-risk cardiac illness during their registration based on the mWHO classification of maternal cardiovascular risk (Table 1). Patients belonging to mWHO I and II will be classified as low-risk, and patients belonging to mWHO II-III, III and IV as high-risk groups. More frequent follow up as per existing guidelines [23] will be recommended for high-risk patients including early hospitalization for safe delivery and child birth as appropriate. However, the frequency and components of follow up will be decided as per the discretion of the participating site investigators or according to the institutional protocol of each participating sites.

### Antenatal counseling and management

Antenatal mothers and their families will be counselled about their cardiac condition, its impact on maternal and fetal outcome, the importance of periodic monitoring and pharmacotherapy and the role of healthcare in supporting them through the pregnancy. Termination of pregnancy will be advised for the mWHO IV patients as applicable. However, if the patient chooses to continue the pregnancy against the medical advice, she will be under close monitoring and supervision throughout pregnancy. Details of optimisation of cardiac medications, safe delivery and childbirth in high-risk patients, anticoagulant management of patients with prosthetic valve in the first trimester, management of complications, interventional procedures like valvotomy will be documented.

### Peripartum management and counselling and discharge

The intra and postpartum management will be taken care by the respective pregnancy and heart teams. Details of the mode of delivery, the type of anesthesia/analgesia, outcome, complications, and management will be documented for all the patients. Patients will undergo a detailed clinical examination and echocardiographic assessment before discharge. Counselling provided will include discussions on need and timing of further follow up, cardiac medications, appropriate contraceptive methods, spacing, breast feeding etc.

### Post-partum care and follow up

**Fig. 2:**
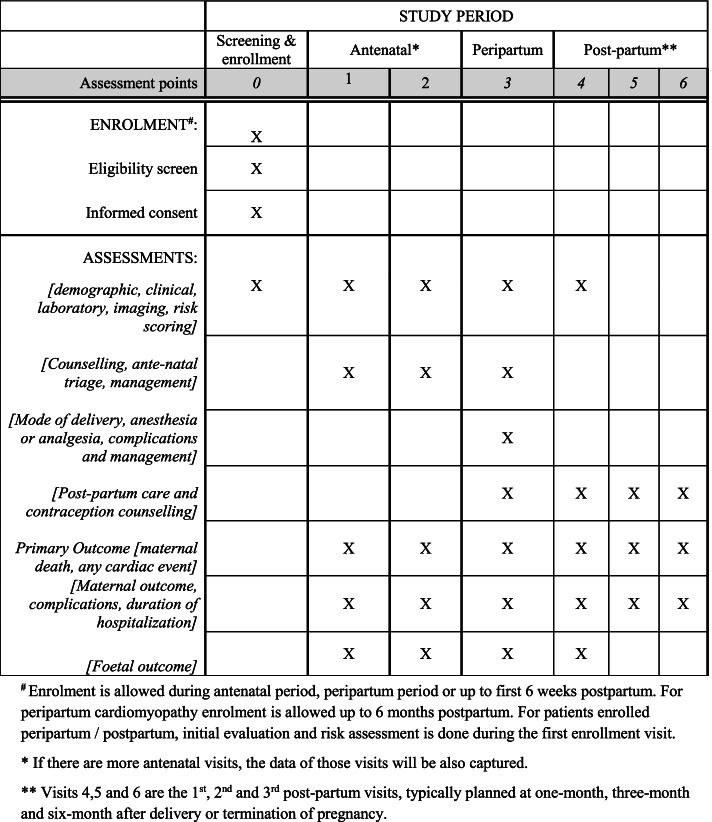
The process of TNPHDR. This figure illustrates the typical process of how a patient is enrolled in the TNPHDR and is followed up till completion of the data collection at 6 months post termination

All patients enrolled in the TNPHDR will be actively followed up for a period of 6 months from the date of delivery/termination of pregnancy. During this time data will be collected at one month, three-month and six-month time points either during the clinical visits or by telephonic calls. At least one clinical follow up including an echocardiographic assessment is mandatory during follow up (Fig. 2).

### Trainings and sensitizations

Initial sensitization cum training program about the TNPHDR and about pregnancy and heart team was conducted for all the site investigators in December 2019. In addition, periodic refresher trainings will be done at the individual sites. The site investigators are encouraged to train their team on data collection and entry in the online platform. Online pregnancy and heart team groups have been formed for each site, which also includes members from the central coordinating nodal team, to discuss any queries on patient management and data entry.

## Outcome measures

Primary outcome will be a composite of maternal cardiac events. Cardiac death, resuscitated cardiac arrest, cardiac hospitalization, new or worsening heart failure requiring treatment escalation or hospitalization, new episode of arrhythmia requiring treatment, thromboembolic event, hemorrhagic complications, aortic dissection, endocarditis, acute coronary syndrome, or cardiac intervention during pregnancy and up to 1 week post-delivery will be included as maternal cardiac events. Individual components of composite primary outcome and all-cause mortality will be the secondary outcomes.

We will include both obstetric and fetal outcomes as additional secondary outcomes. Obstetric outcome measures will include gestational diabetes mellitus, gestational hypertension (at least two blood pressure readings above 140/90 measured more than 6 h apart, after 20 weeks of gestation), pre- eclampsia (gestational hypertension with > 0.3 g proteinuria in the 24 h urine sample), eclampsia (pre-eclampsia with seizures) / HELLP syndrome (haemolysis, elevated liver enzymes, low platelet count), preterm labour (spontaneous onset of labour < 37 weeks of gestation), premature rupture of membranes (membrane rupture before onset of uterine contractions) and mode of delivery. Fetal loss (abortions defined as fetal death < 20 weeks, intra-uterine death defined as fetal death > 20 weeks and still births) will be the main fetal outcome considered in the study. We will also include a composite of fetal loss, premature birth before 37 weeks of gestation, intra-uterine growth retardation or small-for-gestational-age (birth weight < 10th percentile for gestational age or less than 2.5 kg) and congenital heart disease or anomalies in the newborn as additional secondary fetal outcome.

### Data management and analysis

Data uploaded in the TNPHDR server will be verified for accuracy and completeness by the nodal coordinating centre team. Regular assessment will be done to check the appropriateness and completeness of the uploaded data. As required by the International Conference on Harmonisation Good Clinical Practice guidelines (ICH-GCP), the investigator will allow direct access to all registry related medical records to allow the verification of data gathered in the eCRFs and for the review of the data collection process*.* The nodal center staff will conduct periodic site visits to all participating centers to verify the data by random cross-check of the source documents (10% of all data fields), as part of quality assurance. We will use appropriate summary statistics to present the descriptive data. Multivariate regression models will be employed to study predictors of maternal and fetal outcomes.

### Ethical considerations

The TNPHDR follows the ethical principles in line with the current declaration of Helsinki and ‘ethical guidelines for bio- medical research on human participants’ as laid down by the Indian Council of Medical Research (ICMR). Approval for participation in the study was obtained from the Institutional Ethics Committees of the coordinating center and all the participating institutions. The TNPHDR has been registered in the Clinical Trial Registry of India (CTRI/2020/01/022736) and all relevant data will be available in http://www.ctri.nic.in. Information and consent forms will be provided to study participants in a clear, and simple local language. Written informed consent in vernacular will be obtained before enrolment into the registry. All efforts will be made to ensure confidentiality, anonymity and privacy of the information and this shall be assured to all participants and hospital administration.

## Study Status

The study started enrolling patients from 15^th^ January 2020. As of 5^th^ November 2021, 2461 eligible patients have been enrolled in TNPHDR. Some participating sites have stopped enrolling, while the other participating sites and nodal site are continuing enrolment. Antenatal observation and active post-natal follow up are ongoing. The 6-month post-natal follow up data collection of all enrolled patients is expected to be completed by early 2023.

## Discussion

There is limited data on management of PWWHD from LMICs particularly from Asia. The available studies are small, often retrospective, and limited to small geographic areas. The TNPHDR will add large scale prospective data from a LMIC settings, on pre-pregnancy counselling, fetal and maternal outcomes, and outcome predictors in PWWHD. It will give information on applicability of existing risk scoring systems based on data from high-income countries to PWWHD in LMICs.

The data from this study will be used to bring out a management guideline for PWWHD in LMICs. Identifying the gaps in the existing health care delivery, may facilitate initiation of steps for capacity building and empowerment of health care workers and institutions to ensure delivery of quality cardiac care to antenatal mothers with heart disease and formulation of a practical and applicable guideline for management of PWWHD.

## Supplementary Information

**Additional file 1:** List of participating sites with investigators

**Additional file 2:** Case report form booklet

**Additional file 3:** Standard operating procedure

## Data Availability

Data sharing is not applicable to this article, as it is a protocol paper. Once the study is completed and after the publication of the preliminary results, the data will be made available to interesting researchers upon submission of a formal proposal to the corresponding author. The permission of the Institute Ethics Committee of Madras Medical College, Chennai will be obtained prior to the release of deidentified data.

## Abbreviations

AVA: Aortic Valve Area
AV: Atrio Ventricular valve
CHD: Congenital Heart disease
CRF: Case Report Form
HF: Heart Failure
ICH-GCP: International Conference on Harmonisation—Good Clinical Practice guidelines
ICMR: Indian Council of Medical Research
LMIC: Low- and Middle-Income Countries
LV: Left Ventricle
MMR: Maternal Mortality Ratio
PWWHD: Pregnant Women with Heart Disease
RVSP: Right Ventricular systolic pressure
SOP: Standard Operating Procedure
TIA: Transient Ischemic Attack
TNPHDR: Tamil Nadu Pregnancy and Heart Disease Registry
WHO: World Health Organization
WWHD: Women With Heart Disease
mWHO: Modified World Health Organization classification of maternal cardiovascular risk

## References

[1] Cardiac Disease and Pregnancy (Good Practice No. 13) [Internet]. Royal College of Obstetricians & Gynaecologists. [cited 2021 Apr 16]. Available from: https://www.rcog.org.uk/en/guidelines-research-services/guidelines/good-practice-13/

[2] Roberts WT, Adamson D (2013). Cardiovascular disease in pregnancy. Obstet Gynaecol Reprod Med..

[3] Mocumbi A, Sliwa K, Soma-Pillay P (2016). Medical disease as a cause of maternal mortality: the pre-imminence of cardiovascular pathology. Cardiovasc J Afr..

[4] Vangen S, Bødker B, Ellingsen L, Saltvedt S, Gissler M, Geirsson RT (2017). Maternal deaths in the Nordic countries. Acta Obstet Gynecol Scand..

[5] Creanga AA, Syverson C, Seed K, Callaghan WM (2017). Pregnancy-Related Mortality in the United States, 2011–2013. Obstet Gynecol..

[6] MBRRACE KM. MBRRACE-UK: Saving Lives, Improving Mothers’ Care 2020 [Internet]. Birth Companions. [cited 2021 Apr 21]. Available from: /resources/244-mbrrace-uk-saving-lives-improving-mothers-care-2020

[7] Souza JP, Tunçalp Ö, Vogel JP, Bohren M, Widmer M, Oladapo OT (2014). Obstetric transition: the pathway towards ending preventable maternal deaths. BJOG Int J Obstet Gynaecol..

[8] Gnanaraj JP, Princy SA, Surendran SA (2021). Counselling and pregnancy outcomes in women with congenital heart disease- current status and gap analysis from Madras Medical College Pregnancy And Cardiac disease (M-PAC) registry. Int J Cardiol Congenit Heart Dis.

[9] French Katharine A (2018). Poppas Athena. Rheumatic Heart Disease in Pregnancy. Circulation..

[10] Dawson AJ, Krastev Y, Parsonage WA, Peek M, Lust K, Sullivan EA (2018). Experiences of women with cardiac disease in pregnancy: a systematic review and metasynthesis. BMJ Open.

[11] Sullivan EA, Vaughan G, Li Z, Peek MJ, Carapetis JR, Walsh W (2020). The high prevalence and impact of rheumatic heart disease in pregnancy in First Nations populations in a high-income setting: a prospective cohort study. BJOG Int J Obstet Gynaecol..

[12] Millennium Development Goals Report 2015 [Internet]. 2015 [cited 2021 Apr 16]. Available from: mdg-report-2015.html

[13] know India. Home | Know India: National Portal of India [Internet]. [cited 2021 Apr 16]. Available from: https://knowindia.gov.in/

[14] Registrar General SRS RK. SAMPLE REGISTRATION SYSTEM Bulletin on MMR in India. 2020 Jul;4

[15] Heemelaar S, Petrus A, Knight M, Akker T (2020). Maternal mortality due to cardiac disease in low- and middle-income countries. Trop Med Int Health..

[16] tnhealth. Health & Family Welfare Department, Government of Tamil Nadu [Internet]. [cited 2021 Apr 16]. Available from: https://tnhealth.tn.gov.in/tngovin/dfw/dfw.php

[17] Regitz-Zagrosek V, Roos-Hesselink JW, Bauersachs J, Blomström-Lundqvist C, Cífková R, De Bonis M (2018). ESC Guidelines for the management of cardiovascular diseases during pregnancy: The Task Force for the Management of Cardiovascular Diseases during Pregnancy of the European Society of Cardiology (ESC). Eur Heart J..

[18] Thorne S, MacGregor A, Nelson-Piercy C (2006). Risks of contraception and pregnancy in heart disease. Heart Br Card Soc..

[19] Regitz-Zagrosek V, Lundqvist CB, Borghi C, Zagrosek, Association for European Paediatric Cardiology (AEPC), German Society for Gender Medicine (DGesGM) (2011). ESC Guidelines on the management of cardiovascular diseases during pregnancy: the Task Force on the Management of Cardiovascular Diseases during Pregnancy of the European Society of Cardiology (ESC). Eur Heart J.

[20] Siu SC, Sermer M, Colman JM, Alvarez AN, Mercier L-A, Morton BC, et al. Prospective Multicenter Study of Pregnancy Outcomes in Women With Heart Disease. 7 10.1161/hc3001.093437 11479246

[21] Silversides CK, Grewal J, Mason J, Sermer M, Kiess M, Rychel V (2018). Pregnancy Outcomes in Women With Heart Disease. J Am Coll Cardiol.

[22] Drenthen W, Pieper PG, Roos-Hesselink JW, van Lottum WA, Voors AA, Mulder BJM (2007). Outcome of Pregnancy in Women With Congenital Heart Disease. J Am Coll Cardiol.

[23] American College of Obstetricians and Gynecologists' Presidential Task Force on Pregnancy and Heart Disease and Committee on Practice Bulletins—Obstetrics (2019). ACOG Practice Bulletin No. 212: Pregnancy and Heart Disease. Obstet Gynecol.

