# Supplementary material for: Tamil Nadu Pregnancy and Heart Disease Registry (TNPHDR): design and methodology
Source: BMC Pregnancy Childbirth. 2022 Jan 29;22:80. doi: 10.1186/s12884-021-04305-3 (PMC8801092; doi:10.1186/s12884-021-04305-3)
Supplement: Supplementary file 1 — Additional file 1: List of participating sites with investigators [file 12884_2021_4305_MOESM1_ESM.doc]

| **List of sites participating in the Tamil Nadu Pregnancy and Heart Disease Registry** | | | | |
| --- | --- | --- | --- | --- |
| **No** | **Name of the centre** | **Site Principal Investigator Cardio** | **Site Principal Investigator OBG** | **Role** |
| 1 | Rajiv Gandhi Government General Hospital , MMC, Chennai | Dr.G Justin Paul (Inv)  Dr. E.Elavarasi (Co-Inv) | Dr. S. Vijaya | State coordinating / Nodal centre & Participating centre |
| 2 | IOG, MMC, Chennai | Dr. S.Anne Princy | Dr.S.Gomathy | Participating centre |
| 3 | TNGMSSH, KGH Omandurar Medical College | Dr. Cecily Mary Majella | Dr.Malarvizhi K.L. | Participating centre |
| 4 | RSRM, Stanley Medical College, Chennai | Dr.Kumaresan Kannan | Dr. R. Venkadeshwari | Participating centre |
| 5 | Kilpauk Medical College, Chennai | Dr. M. Nandakumaran | Dr.Vanitha | Participating centre |
| 6 | Chengalpattu Medical College | Dr.Ragothaman. S | Dr.Thenmozhi | Participating centre |
| 7 | Vellore Medical College | Dr.Sabapathy. K | Dr.Pushpalatha | Participating centre |
| 8 | Tiruvannamalai Medical College | Dr. R. Kannan | Dr. J. Arumai Kannu | Participating centre |
| 9 | Villupuram Medical College | Dr.Kathirvel | Dr.Rajeshwari | Participating centre |
| 10 | Dharmapuri Medical College | Dr. B. Kannan | Dr. L. Malarvizhi | Participating centre |
| 11 | GMKM Medical College, Salem | Dr.Kannan | Dr.Subha Periasamy | Participating centre |
| 12 | Karur Medical College | Dr.Sushil Kumar. E | Dr. S. Gayathri Devi | Participating centre |
| 13 | Government KAPV Medical College, Trichy | Dr.Balasubramanian | Dr. Uma | Participating centre |
| 14 | Coimbatore Medical College | Dr.Nambirajan Jeyapalan | Dr. R. Manonmani | Participating centre |
| 15 | Tiruvarur Medical College | Dr.Chackaravarthy .P.S | Dr. A. Prabha | Participating centre |
| 16 | Thanjavur Medical College | Dr. T. Gomathi | Dr. R. Rajarajeshwari | Participating centre |
| 17 | Pudukkottai Medical College | Dr.Nachiappan. K | Dr. P. Amudha | Participating centre |
| 18 | Madurai Medical College | Dr.Veeramani | Dr. N. Sumathi | Participating centre |
| 19 | Sivagangai Medical College | Dr. R. Shankar | Dr.Gayathri | Participating centre |
| 20 | Theni Medical College | Dr.Aravazhi | Dr.B.Shanthi Rani | Participating centre |
| 21 | Tirunelveli Medical College | Dr. Ravi Edwin | Dr.Ramalashmi | Participating centre |
| 22 | Thoothukudi Medical College | Dr. S. Ganesan | Dr. P. Muthu Prabha | Participating centre |
| 23 | Kanyakumari Medical College | Dr.Muralitharan | Dr.Sundaravani | Participating centre |
| 24 | Dr.Mehta Hospital, Chennai | Dr.Prabhakar Dorairaj | Dr.Nandita Thakkar | Participating centre |
| 25 | Sri Ramachandra Medical College | Dr. S.Sadanantham | Dr.Dhanalakshmi | Participating centre |
| 26 | Apollo first med Hospitals | Dr.Prabhakar Dorairaj | Dr.Soundarya Priya | Participating centre |
| 27 | Madras Medical Mission | Dr.Ramkumar | Dr.Rashmi Gingade Vittal | Participating centre |
| 28 | Christian Medical College, Vellore | Dr. Paul George | Dr.Sowmya Sathyendra | Participating centre |
| 29 | Velammal Medical College | Dr.R Vadivelu | Dr. K. Sasikala | Participating centre |
