# Supplementary material for: Tamil Nadu Pregnancy and Heart Disease Registry (TNPHDR): design and methodology
Source: BMC Pregnancy Childbirth. 2022 Jan 29;22:80. doi: 10.1186/s12884-021-04305-3 (PMC8801092; doi:10.1186/s12884-021-04305-3)
Supplement: Supplementary file 2 — Additional file 2: Case report form booklet [file 12884_2021_4305_MOESM2_ESM.pdf]

TAMIL NADU PREGNANCY & HEART DISEASE REGISTRY

DATA COLLECTION FORM

Name of the institution :

Filled by :

Department :

Signature :

<u>சுய ஒப்புதல் படிவம்</u>

தமிழ்நாடு கர்ப்பம் மற்றும் இதய நோய் பதிவு

ஆராய்ச்சி நிலையம்: இருதய மருத்துவ நிறுவனம்,
இராஜீவ் காந்தி அரசு பொது மருத்துவமனை மற்றும்
சென்னை மருத்துவக்கல்லூரி,
சென்னை - 600 003.

பங்கு பெறுபவர் இதனை (✓) குறிக்கவும்

மேலே குறிப்பிட்டுள்ள மருத்துவ ஆய்வின் விவரங்கள் எனக்கு விளக்கப்பட்டது. என்னுடைய சந்தேகங்களைக் கேட்கவும், அதற்கான தகுந்த விளக்கங்களைப் பெறவும் வாய்ப்பளிக்கப்பட்டது. ☐

நான் இவ்வாய்வில் தன்னிச்சையாகத்தான் பங்கேற்கிறேன். எந்தக் காரணத்தினாலோ எந்தக் கட்டத்திலும், எந்த சட்டசிக்கலுக்கும் உட்படாமல் நான் இவ்வாய்வில் இருந்து விலகிக் கொள்ளலாம் என்றும் அறிந்துகொண்டேன். ☐

இந்த ஆய்வு சம்மந்தமாகவும், மேலும் இது சார்ந்த ஆய்வு மேற்கொள்ளும் போதும், இந்த ஆய்வில் பங்கு பெறும் மருத்துவர் என்னுடைய மருத்துவ அறிக்கைகளைப் பார்ப்பதற்கு என் அனுமதி தேவையில்லை என அறிந்து கொள்கிறேன். ☐

நான் ஆய்வில் இருந்துவிலகிக் கொண்டாலும் இது பொருந்தும் என அறிகிறேன். ☐

இந்த ஆய்வின் மூலம் கிடைக்கும் தகவல்களையும், பரிசோதனை முடிவுகளையும் மற்றும் சிகிச்சை தொடர்பான தகவல்களையும் மருத்துவர் மேற்கொள்ளும் ஆய்வில் பயன்படுத்திக்கொள்ளவும், அதைப் பிரசுரிக்கவும் என் முழு மனதுடன் சம்மதிக்கிறேன். ☐

இந்த ஆய்வில் பங்கு கொள்ள ஒப்புக்கொள்கிறேன். எனக்குக் கொடுக்கப்பட்ட அறிவுரைகளின் படி நடந்து கொள்வதுடன், இந்த ஆய்வை மேற்கொள்ளும் மருத்துவ அணிக்கு உண்மையுடன் இருப்பேன் என்றும் உறுதியளிக்கிறேன். ☐

என் உடல் நலம் பாதிக்கப்பட்டாலோ அல்லது எதிர் பாராத வழக்கத்திற்கு மாறாக நோய்க்குறி தென்பட்டாலோ உடனே அதை மருத்துவ அணியிடம் தெரிவிப்பேன் என உறுதி அளிக்கிறேன். ☐

இந்த ஆய்வில் எனக்கு இரத்தப் பரிசோதனை மற்றும் இதர மருத்துவப் பரிசோதனைகள் செய்து கொள்ள நான் முழுமனதுடன்சம்மதிக்கிறேன். ☐

இந்த ஆய்வில் என் தகவல்களை மாநில மற்றும் தேசிய மருத்துவ நலனுக்காக பயன்படுத்திக் கொள்ள என் முழு மனதுடன் சம்மதிக்கிறேன். ☐

பங்கு பெறுபவரின் எண்:

பங்கேற்பவரின் கையொப்பம் .... இடம் .... தேதி ....

கட்டைவிரல் ரேகை:

பங்கேற்பவரின் பெயர் மற்றும் விலாசம் ....

ஆய்வாளரின் கையொப்பம் .... இடம் ....தேதி ....

ஆய்வாளரின் பெயர் ....

A1. TNPHDR No: __ __ __ __ / __ __ __ / __ __ __ / __ __ ____ __

A2. RCH. No :__ __ __ __ __ __ __ __ __ __ __ __ __

A3. DATE OF REGISTRATION: _____ /_____ / _________

TAMIL NADU PREGNANCY AND HEART DISEASE REGISTRY-DATA COLLECTION FORM

INCLUSION CRITERIA CHECKLIST*

For all patients* (Tick the applicable)	Yes	No	A4. ◯ Antenatal<br>◯ Post-partum (Up to 6 wks)<br>◯ Postnatal (1-5 months, only for peri-partum cardiomyopathy)
	Yes	No	A5. Heart disease
	Yes	No	A6. Consented for the study

*For all registered patients, the above three rows should be Yes

BASELINE DATA- IDENTIFIER PAGE

**B1**	Hospital/PIN number*:		
**B2**	Full name of the patient (IN BLOCK LETTERS)*:		
**B3**	Name of the Husband/Guardian*:		
**B4**	Age *(in completed years):* Y Y	**B5** Date of Birth*:	D D M M Y Y Y Y
**B6**	House/Flat name or number*:		
**B7**	Street/locality*:		
**B8**	State*:		
**B9**	District*:		
**B10**	Taluk*:	**B11** Village*:	
**B12**	Post office*:	**B13** Pin code*	
**B14**	Aadhaar no:		
**B15**	Patient's mobile number*	0	
**B16**	Patient's alternate mobile number	0	
**B17**	Relative's mobile number	0	
	Name of the relative:	Relationship:	
**B18**	Total number of years of education* (Zero for Illiterates) Y Y	Qualification (Enter the highest degree)<br>◯ Uneducated ◯ Primary school ◯ Middle school<br>◯ High school ◯ Higher Secondary ◯ Undergraduate<br>◯ Postgraduate ◯ Doctorate	
**B19**	Occupation*:		
**B20**	Marital status*: ◯ Married ◯ Never Married ◯ Widowed ◯ Divorced/ Separated		
**B21**	Total monthly income of the family:	**B22** Total members of the family :	
**B23**	Socio-economic status*: (As per ration card) ◯ Above Poverty Line ◯ Below Poverty Line		

REGISTRATION DETAILS-REGISTRATION PAGE

TIME OF FIRST DIAGNOSIS OF HEART DISEASE *(tick applicable)*

C1 Chronological age at diagnosis *(in years)* : ________

- ○ Before marriage
- ○ After marriage

During present pregnancy ○
Prior to present pregnancy ○

Gestational age at diagnosis (In weeks) : ________

If prior to present pregnancy, specify whether during previous pregnancy ○Yes ○ No

PRE-PREGNANCY DETAILS *(tick applicable)*

C2 Pre-pregnancy cardiac and related history

C2.1 NYHA CLASS ○ Class I ○ Class II ○ Class III ○ Class IV

**C2.2** Prior HF	**C2.3** Prior AF	**C2.4** Prior procedures	**C2.5** Prior OAC use	**C2.6** Others
☐	☐	☐	☐	☐

C2. 7 Details of prior procedures done (Surgical procedures or Cardiac interventions)

Name of the procedure	
Chronological age during the procedure (in yrs)	
Institution where intervention was done	

C2. 8 Prior Hospitalisations (if any) ○Yes ○No
Reason for hospitalization: ____________
____

Number of days hospitalized: | M | M |

Month & year of hospitalization: | M | M | Y | Y | Y | Y |

C2.9 Medications on: *(specify if on any drugs with dosage)*

**C2.10** Diabetes	**C2.11** SHT	**C2.12** CAD	**C2.13** Alcohol	**C2.14** Smoking	**C2.15** Tobacco use
☐	☐	☐	☐	☐	☐

C2.16 ☐ Others, Specify

PREVIOUS PREGNANCY DETAILS ○Yes ○No ***(In case of more than one previous pregnancy kindly attach a copy of this page for each)***

C3

C3.1 Date of delivery/termination of previous pregnancy | D | D | M | M | Y | Y | Y | Y |

C3.2 Gestational age at delivery (weeks) | | |

C3.3 Mode of delivery ○ Vaginal ○ Assisted vaginal ○ LSCS

C3.4 Foetal outcome
○ Live birth ○ IUD ○ Still birth
○ Spontaneous Abortion ○ Induced Abortion
Birth Weight(In Kgs) | | . | | | |

C3.5 AN/PN decompensation ○ Yes ○ No
If yes details:
- ☐ Heart failure
- ☐ Cardiogenic shock
- ☐ Thrombotic complications
- ☐ Others(specify)
- ☐ Bleeding complications
- ☐ Arrhythmia
- ☐ Infective complications(endocarditis)

C3. 6 Any antenatal interventions done ○ Yes ○ No *(If yes, Kindly specify)*

Name of the procedure	
Gravida when procedure done	
Gestational age during procedure	
Institution where done	

C3.7 Post-partum interventions (within 6weeks of delivery). ○ Yes ○ No *(If yes, Kindly specify)*

Name of the procedure	
Post-natal day procedure done	
Institution where done	

C3.8 Was the previous pregnancy continued Against Medical Advice ○ Yes ○ No

TNPHDR

PRESENT PREGNANCY DETAILS		
	**ANTENATAL DETAILS**	
**C4**	Obstetric score	G | P | L | A ○ Singleton pregnancy ○ Twin pregnancy ○ Others
**C5**	LMP (Last Menstrual Period)	D D M M Y Y Y Y
**C6**	EDD (Expected Date of Delivery)	D D M M Y Y Y Y
**C7**	Gestational age at registration (weeks)	
**C8**	Antenatal check-ups (tick applicable)	
	**C8. 1** ○ Booked ○ Unbooked ○ Status not known	
	**C8. 2** Institution where AN check-up done:	
**C9**	**ASSOCIATED RISK FACTORS** *(tick all applicable)*	

**C9.1** PIH	**C9.2** GDM	**C9.3** Hypothyroid	**C9.4** Hyperthyroid	**C9.5** Anaemia	**C9.6** Rheumatologic problems
☐	☐	☐	☐	☐	☐

C9.7 ☐ Others, specify

C10 **COMPLETE DIAGNOSIS**

____

____

- ☐ Heart failure
- ☐ Pulmonary hypertension ○ Primary ○ Secondary
- ☐ Atrial fibrillation
- ☐ Rheumatic Heart disease
- ☐ Cardiomyopathy
- ☐ Aortopathy/ Diseases of Aorta
- ☐ Congenital Heart disease
- ☐ Arrhythmia
- ☐ Coronary artery disease
- ☐ Post-Interventional. If yes, Specify ________________

C11 Present pregnancy continued Against Medical Advice ○ Yes ○ No

C12 Any antenatal interventions done before registration ○ Yes ○ No *(If yes, Kindly specify)*

Name of the procedure	
Gravida when procedure done	
Gestational age during procedure	
Institution where done	

C13 Post-partum interventions (Only for cases registered in TNPHDR within 6weeks of delivery) ○ Yes ○ No *(If yes, specify)*

Name of the procedure	
Post-natal day procedure done	
Institution where done	

TNPHDR

FIRST VISIT PAGE

Date | D | D | M | M | Y | Y | Y | Y |

D1 PATIENT SYMPTOMS

D1.1 NYHA CLASS ○ Class I ○ Class II ○ Class III ○ Class IV

Presenting symptoms (tick all applicable)

**D1.2** Nil	**D1.3** Dyspnea	**D1.4** PND	**D1.5** Orthopnea	**D1.6** Palpitation	**D1.7** Pedal edema	**D1.8** Chest pain	**D1.9** Syncope
☐	☐	☐	☐	☐	☐	☐	☐

D1.10 ☐ **Others** *(Specify):*

D2 CLINICAL SIGNS

**D2.1** Ht (cm)		**D2.2** Wt (Kg)		**D2.3** RR (/min)	
**D2.4** HR (/min)		**D2.5** BP(mm Hg)		**D2.6** SPO2 (%)	

D2.7 CVS: S1______________ S2______________

Murmur: ○ Yes ○ No ☐ Sys ☐ Dias

Gallop: ○ Yes ○ No ☐ LVS3 ☐ RVS3

D2.8 JVP ○ Normal ○ Elevated

D2.9 RS ○ Normal ○ Abnormal ☐ Crepitations ☐ Rhonchi/ Wheeze

D3 BASELINE INVESTIGATIONS:

D3.1 Blood Sugar (mg/dl) ☐☐☐

D3.2 Blood Urea (mg/dl) ☐☐☐

D3.3 Sr. Creatinine (mg/dl) ☐ . ☐☐

D3.4 Hb (g/dl) ☐☐ . ☐

D3.5 Others Relevant investigations ☐☐☐☐

D3.6 ECG ○ Normal ○ Abnormal. If abnormal, Choose:

☐ AF ☐ SVT ☐ VT ☐ 1°AVB ☐ 2 AVB ☐ CHB ☐ ST↑ ☐ ST↓ ☐ **Others**

Others, Specify:

D4 RISK ASSESSMENT AND TRIAGE

D4.1 Modified WHO Risk assessment Scale: ○ m WHO I ○ m WHO II ○ m WHO II-III ○ m WHO III ○ m WHO IV

D4.2 CARPREG SCORE: *(Tick the appropriate box)*

S. No	Patient Variable	Yes	No
1	Prior cardiac event [Heart Failure, Stroke, TIA before pregnancy, arrhythmia]		
2	Baseline NYHA III / IV or Cyanosis		
3	Left Heart Obstruction [MVA < 2sq.cm, AVA < 1.5 sq.cm, Peak LVOT gr > 30 mm Hg		
4	Reduced systemic ventricular systolic function < 40%		

D4.3 CARPREG II SCORE: *(Tick the appropriate box)*

S. No	Patient Variable	Yes	No
1	Prior cardiac event/ arrhythmia		
2	Baseline NYHA III / IV or Cyanosis		
3	Mechanical prosthetic valves		
4	Ventricular dysfunction		
5	High Risk Lt sided valve disease, LVOTO		
6	Pulmonary Hypertension		
7	CAD		
8	High risk aortopathy		
9	No Prior cardiac interventions		
10	Late pregnancy assessment		

D4.4 ZAHARA SCORE: *(Tick the appropriate box)*

S. No	Patient Variable	Yes	No
1	History of arrhythmic events		
2	Baseline NYHA III or IV		
3	LVOT obstruction >50 mm Hg		
4	Mechanical valve prosthesis		
5	Cardiac drugs pre-pregnancy use		
6	CCHD [repaired or unrepaired]		
7	Moderate to severe sub pulmonic AV valve regurgitation		
8	Moderate to severe systemic AV valve regurgitation		

D5	**ECHOCARDIOGRAPHIC ASSESSMENT** ○ **Normal** ○ **Abnormal**

**D5.1 Situs** ○ Solitus ○ Inversus† ○ Ambiguus†	**D5.2 Position** ○ Dextrocardia† ○ Levocardia		
**D5.3 AV concordance** ○ Concordant ○ Discordant†	**VA concordance** ○ Concordant ○ Discordant†		

D5.4 Chamber description

Morphological LA	Normal: ○ Yes ○ No	○ Dilated	○ Hypoplastic†	***# Kindly fill form I***
Morphological RA	Normal: ○ Yes ○ No	○ Dilated	○ Hypoplastic†	***† Kindly fill Form J***
Morphological LV	Normal: ○ Yes ○ No	○ Dilated	○ Hypoplastic†	***¥ Kindly fill form K***
Morphological RV	Normal: ○ Yes ○ No	○ Dilated	○ Hypoplastic†	***Kindly fill form L***

D5.5 Ventricular function

Wall Motion: ○ Normal ○ Regional wall motion abnormalities ○ Global Hypokinesia ¥

LV systolic function	LVID	Diastole(mm)		EF and LV function	
		Systole(mm)		>54%	○ Normal ¥
	EF%			40-54%	○ Mild LV systolic dysfunction¥
LV diastolic function		E(cm/s)		30-40%	○ Moderate LV systolic dysfunction¥
		A(cm/s)		<30%	○ Severe LV systolic dysfunction¥
RV systolic function ○Normal ○Abnormal		TAPSE (mm)		Sa'	

D5.6 Valve morphology and function

Mitral	○ Native<br>○ Prosthetic#	Structure ○Normal ○Abnormal<br>☐ Prolapse ☐ Thickened<br>☐ Degenerative ☐ Flial	Function ○Normal ○Abnormal<br>☐ Stenotic ○ Mild ○ Moderate ○ Severe<br>MVOA____________________Cm²<br>MV Gradient: Mean_________ Peak____________<br>☐ Regurgitant ○ Mild ○ Moderate ○ Severe
Aortic	○ Native<br>○ Prosthetic#	Structure ○Normal ○Abnormal<br>☐ Prolapse ☐ Thickened<br>☐ Degenerative ☐ Calcific	Function ○Normal ○Abnormal<br>☐ Stenotic^Δ ○Mild ○ Moderate ○ Severe<br>Gradient: Mean_________ Peak____________<br>☐ Regurgitant ○Mild ○Moderate ○ Severe
Tricuspid	○ Native<br>○ Prosthetic#	If native<br>○ Normal<br>○ Prolapse	Function ○ Normal ○ Abnormal<br>☐ Stenotic ○Mild ○Moderate ○Severe<br>Gradient: Mean_________ Peak____________<br>☐ Regurgitant ○Mild ○ Moderate ○ Severe
Pulmonary	○ Native<br>○ Prosthetic#	If native<br>○ Normal ○ Absent<br>○ Dooming	Function ○ Normal ○ Abnormal<br>☐ Stenotic^Δ ○Mild ○Moderate ○ Severe<br>Gradient: Mean_________ Peak____________<br>☐ Regurgitant ○Mild ○Moderate ○ Severe

D5.7 Pulmonary pressures

Tricuspid regurgitation-TRPG(mmHg): | Pulmonary regutgitation-Peak PR(mmHg):

**Grading of PHT**	☐ Nil	☐ Mild PHT	☐ Moderate PHT	☐ Severe PHT
TPRG	<30	30-45	45-60	>60
Peak PR	<15	15-35	35-45	>45

**D5.8 IAS**	○ Intact ○ Defective†
**D5.9 IVS**	○ Intact ○ Defective†
**D5.10 Other Shunts†**	☐ PDA ☐ APW ☐ RSOV ☐ Others
**D5.11 Pericardium**	☐ Normal ☐ Thickened ☐ Effusion ○ Mild ○ Moderate ○ Massive
**D5.12 Aorta**	○ Normal ○ Abnormal. If Abnormal, Specify ○ Dilated ○ Coarctation ○ Others
**D5.13 Others**	☐ Vegetations ☐ Thrombus

**D6**	TNPHDR Risk category (as per triage): ○ LOW RISK ○ HIGH RISK
**D7**	Specific Advice in the visit(if any) : ☐ Nil ☐ Hospitalization ☐ Change of medication<br>☐ Advice intervention ○ Cardiac ○ Others, specify__________
**D8**	Date of next follow up: D D M M Y Y Y Y

E. DRUG PAGE-USE AND DOSAGE

(tick the relevant drugs and mention the dosage used)

NO	DRUG	E1 PRE- PREGNANCY		E2 I TRIMESTER		E3 II TRIMESTER		E4 III TRIMESTER		E5 PERIPARTUM	
			DOSAGE		DOSAGE		DOSAGE		DOSAGE		DOSAGE
1	Penicillin	☐		☐		☐		☐		☐	
2	Warfarin	☐		☐		☐		☐		☐	
3	Acenocoumarol	☐		☐		☐		☐		☐	
4	Phenindione	☐		☐		☐		☐		☐	
5	Aspirin	☐		☐		☐		☐		☐	
6	Clopidogrel	☐		☐		☐		☐		☐	
7	Nitrates	☐		☐		☐		☐		☐	
8	Atorvastatin	☐		☐		☐		☐		☐	
9	Rosuvastatin	☐		☐		☐		☐		☐	
10	Verapamil	☐		☐		☐		☐		☐	
11	Nifedipine	☐		☐		☐		☐		☐	
12	Diltiazem	☐		☐		☐		☐		☐	
13	Atenolol	☐		☐		☐		☐		☐	
14	Metoprolol	☐		☐		☐		☐		☐	
15	Other BBs	☐		☐		☐		☐		☐	
16	Digoxin	☐		☐		☐		☐		☐	
17	Frusemide	☐		☐		☐		☐		☐	
18	Spironolactone	☐		☐		☐		☐		☐	
19	Hydrochlorothiazide	☐		☐		☐		☐		☐	
20	Enalapril	☐		☐		☐		☐		☐	
21	Ramipril	☐		☐		☐		☐		☐	
22	Other ACEIs	☐		☐		☐		☐		☐	
23	Losartan	☐		☐		☐		☐		☐	
24	Telmisartan	☐		☐		☐		☐		☐	
25	Other ARBs	☐		☐		☐		☐		☐	
26	Sildenafil	☐		☐		☐		☐		☐	
27	Bosentan	☐		☐		☐		☐		☐	
28	Amiodarone	☐		☐		☐		☐		☐	
29	Oral anti-diabetics	☐		☐		☐		☐		☐	
30	Insulin	☐		☐		☐		☐		☐	
31	Other drugs	☐		☐		☐		☐		☐	

SECOND VISIT PAGE

Date: D D M M Y Y Y Y

F1 PATIENT SYMPTOMS

F1.1 NYHA CLASS ○ Class I ○ Class II ○ Class III ○ Class IV

Presenting symptoms (tick all applicable)

F1.2 Nil	F1.3 Dyspnea	F1.4 PND	F1.5 Orthopnea	F1.6 Palpitation	F1.7 Pedal edema	F1.8 Chest pain	F1.9 Syncope
☐	☐	☐	☐	☐	☐	☐	☐

F1.10 ☐ **Others** *(Specify)*:

F2 CLINICAL SIGNS & ECG

**F2.1** Ht (cm)		**F2.2** Wt (Kg)		**F2.3** RR (/min)	
**F2.4** HR (/min)		**F2.5** BP(mm Hg)		**F2.6** SPO2 (%)	

F2.7 CVS: S1_____________ S2_____________
Murmur: ○ Yes ○ No ☐ Sys ☐ Dias
Gallop: ○ Yes ○ No ☐ LVS3 ☐ RVS3

F2.8 JVP ○ Normal ○ Elevated

F2.9 RS ○ Normal ○ Abnormal ☐ Crepitations ☐ Rhonchi/ Wheeze

F2.10 ECG ○ Normal ○ Abnormal. If abnormal, Choose:
☐ AF ☐ SVT ☐ VT ☐ 1°AVB ☐ 2 AVB ☐ CHB ☐ ST↑ ☐ ST↓ ☐ **Others**

Others, Specify:

F3 ECHOCARDIOGRAPHIC ASSESSMENT

Ventricular function

Wall Motion: ○ Normal ○ Global Hypokinesia ○ Regional wall motion abnormalities

LV systolic function	LVID	Diastole(mm)	
		Systole(mm)	
	EF%		
LV diastolic function	E(cm/s)		
	A(cm/s)		
RV systolic function	○ Normal ○ Abnormal TAPSE (mm)_________ Sa'_________		

Pulmonary pressures

Tricuspid regurgitation	TRPG (mmHg) :
Pulmonary regurgitation	Peak PR (mmHg):
**Shunt lesion (Nature of shunt)**	○ L→R ○ R→L ○ Bi directional
**Pericardial effusion** *(if present)*	○ Mild ○ Moderate ○ Massive
**Others**	☐ Vegetations ☐ Thrombus

Valve function *(if prosthetic valve kindly fill form I)*

Mitral	☐ Normal	☐ Stenotic ○ Mild ○ Moderate ○ Severe<br>☐ Regurgitant ○ Mild ○ Moderate ○ Severe	MVOA:_____________ cm^2<br>Mean Gradient:<br>Peak gradient:
Aortic	☐ Normal	☐ Stenotic ᐞ ○ Mild ○ Moderate ○ Severe<br>☐ Regurgitant ○ Mild ○ Moderate ○ Severe<br>ᐞ *kindly fill form L*	Mean Gradient:<br>Peak gradient
Tricuspid	☐ Normal	☐ Stenotic ○ Mild ○ Moderate ○ Severe<br>☐ Regurgitant ○ Mild ○ Moderate ○ Severe	Mean Gradient:<br>Peak gradient
Pulmonary	☐ Normal	☐ Stenotic ᐞ ○ Mild ○ Moderate ○ Severe<br>☐ Regurgitant ○ Mild ○ Moderate ○ Severe<br>ᐞ *kindly fill form L*	Mean Gradient:<br>Peak gradient

F4 Specific Advice in the visit (if any) : ☐ Nil ☐ Hospitalization ☐ Change of medication
☐ Intervention ○ Cardiac ○ Others, specify__________
Name of the procedure:
Date : D D M M Y Y Y Y

F5 TNPHDR Risk category (as per triage): ○ LOW RISK ○ HIGH RISK

F6 Date of next follow up: D D M M Y Y Y Y

THIRD VISIT PAGE

Date: D D M M Y Y Y Y

G1 PATIENT SYMPTOMS

G1.1 NYHA CLASS ○ Class I ○ Class II ○ Class III ○ Class IV

Presenting symptoms (tick all applicable)

G1.2 Nil	G1.3 Dyspnea	G1.4 PND	G1.5 Orthopnea	G1.6 Palpitation	G1.7 Pedal edema	G1.8 Chest pain	G1.9 Syncope
☐	☐	☐	☐	☐	☐	☐	☐

G1.10 ☐ **Others** *(Specify):*

G2 CLINICAL SIGNS & ECG

**G2.1** Ht (cm)		**G2.2** Wt (Kg)		**G2.3** RR (/min)	
**G2.4** HR (/min)		**G2.5** BP(mm Hg)		**G2.6** SPO2 (%)	

G2.7CVS: S1_____________ S2______________
Murmur: ○Yes ○ No ☐ Sys ☐ Dias
Gallop: ○ Yes ○ No ☐ LVS3 ☐ RVS3

G2.8 JVP ○ Normal ○ Elevated

G2.9 RS ○ Normal ○ Abnormal ☐ Crepitations ☐ Rhonchi/ Wheeze

G2.10 ECG ○ Normal ○ Abnormal. If abnormal, Choose:
☐ AF ☐ SVT ☐ VT ☐ 1°AVB ☐ 2 AVB ☐ CHB ☐ ST↑ ☐ ST↓ ☐ **Others**

Others, Specify:

G3 ECHOCARDIOGRAPHIC ASSESSMENT

**Ventricular function**				**Pulmonary pressures**	
Wall Motion: ○ Normal ○ Global Hypokinesia ○ Regional wall motion abnormalities				Tricuspid regurgitation	TRPG (mmHg) :
				Pulmonary regurgitation	Peak PR (mmHg):
LV systolic function	LVID	Diastole(mm)		**Shunt lesion (Nature of shunt)**	○ L→R ○ R→L ○ Bi directional
		Systole(mm)			
	EF%			**Pericardial effusion** *(if present)*	○ Mild ○ Moderate ○ Massive
LV diastolic function	E(cm/s)				
	A(cm/s)				
RV systolic function	○Normal ○ Abnormal TAPSE (mm)_________ Sa'______			**Others**	☐ Vegetations ☐ Thrombus

Valve function *(if prosthetic valve kindly fill form I)*

Mitral	☐ Normal	☐ Stenotic ○Mild ○ Moderate ○ Severe<br>☐ Regurgitant ○Mild ○ Moderate ○ Severe	MVOA:______________cm^2<br>Mean Gradient:<br>Peak gradient:
Aortic	☐ Normal	☐ Stenotic ᐞ ○Mild ○ Moderate ○Severe<br>☐ Regurgitant ○ Mild ○ Moderate ○ Severe<br>ᐞ *kindly fill form L*	Mean Gradient:<br>Peak gradient
Tricuspid	☐ Normal	☐ Stenotic ○ Mild ○ Moderate ○ Severe<br>☐ Regurgitant ○ Mild ○ Moderate ○ Severe	Mean Gradient:<br>Peak gradient
Pulmonary	☐ Normal	☐ Stenotic ᐞ ○ Mild ○ Moderate ○ Severe<br>☐ Regurgitant ○ Mild ○ Moderate ○ Severe<br>ᐞ *kindly fill form L*	Mean Gradient:<br>Peak gradient

G4

Specific Advice in the visit (if any) : ☐ Nil ☐ Hospitalization ☐ Change of medication
☐ Intervention ○ Cardiac ○ Others, specify__________

Name of the procedure:
Date : D D M M Y Y Y Y

G5

TNPHDR Risk category (as per triage): ○ LOW RISK ○ HIGH RISK

G6

Date of next follow up: D D M M Y Y Y Y

PERIPARTUM VISIT PAGE

(if the patient is registered for the first time during the peripartum period, then kindly fill first visit page also along with this)

**Date of admission**	D D M M Y Y Y Y	**Date of discharge**	D D M M Y Y Y Y

H1 PATIENT SYMPTOMS

H1.1 NYHA CLASS ○ Class I ○ Class II ○ Class III ○ Class IV

Presenting symptoms (tick all applicable)

**H1.2** Nil	**H1.3** Dyspnea	**H1.4** PND	**H1.5** Orthopnea	**H1.6** Palpitation	**H1.7** Pedal edema	**H1.8** Chest pain	**H1.9** Syncope
☐	☐	☐	☐	☐	☐	☐	☐

H1.10 ☐ Others____________

H2 RISK ASSESSMENT AND TRIAGE

H2.1 Modified WHO Risk assessment Scale: ○ m WHO I ○ m WHO II ○ m WHO II-III ○ m WHO III ○ m WHO IV

H2.2 TNPHDR Risk category (as per triage): ○ LOW RISK ○ HIGH RISK

H3 CLINICAL SIGNS & ECG

**H3.1** Ht (cm)		**H3.2** Wt (Kg)		**H3.3** RR (/min)	
**H3.4** HR (/min)		**H3.5** BP(mm Hg)		**H3.6** SPO2 (%)	

H3.7 CVS: S1______________ S2______________
Murmur: ○ Yes ○ No ☐ Sys ☐ Dias
Gallop: ○ Yes ○ No ☐ LVS3 ☐ RVS3

H3.8 JVP
○ Normal
○ Elevated

H3.9 RS ○ Normal ○ Abnormal
☐ Crepitations
☐ Rhonchi/ Wheeze

H3.10 ECG ○ Normal ○ Abnormal. If abnormal, Choose:

☐ AF ☐ SVT ☐ VT ☐ 1°AVB ☐ 2 AVB ☐ CHB ☐ ST↑ ☐ ST↓ ☐ **Others**

Others, Specify:

H4 ECHOCARDIOGRAPHIC ASSESSMENT

Ventricular function

Wall Motion: ○ Normal ○ Global Hypokinesia ○ Regional wall motion abnormalities

LV systolic function	LVID	Diastole(mm)	
		Systole(mm)	
	EF%		
LV diastolic function	E(cm/s)		
	A(cm/s)		
RV systolic function	○ Normal ○ Abnormal<br>TAPSE (mm)__________ Sa'________		

Pulmonary pressures

Tricuspid regurgitation	TRPG (mmHg) :
Pulmonary regurgitation	Peak PR (mmHg):
**Shunt lesion (Nature of shunt)**	○ L→R ○ R→L<br>○ Bi-directional
**Pericardial effusion** *(if present)*	○ Mild<br>○ Moderate<br>○ Massive
**Others**	☐ Vegetations ☐ Thrombus

Valve function *(if prosthetic valve kindly fill form* ***I*** *)*

Mitral	☐ Normal	☐ Stenotic ○ Mild ○ Moderate ○ Severe<br>☐ Regurgitant ○ Mild ○ Moderate ○ Severe	MVOA:______________cm^2<br>Mean Gradient:<br>Peak gradient:
Aortic	☐ Normal	☐ Stenotic ᐞ ○ Mild ○ Moderate ○ Severe<br>☐ Regurgitant ○ Mild ○ Moderate ○ Severe<br>ᐞ *kindly fill form L*	Mean Gradient:<br>Peak gradient
Tricuspid	☐ Normal	☐ Stenotic ○ Mild ○ Moderate ○ Severe<br>☐ Regurgitant ○ Mild ○ Moderate ○ Severe	Mean Gradient:<br>Peak gradient
Pulmonary	☐ Normal	☐ Stenotic ᐞ ○ Mild ○ Moderate ○ Severe<br>☐ Regurgitant ○ Mild ○ Moderate ○ Severe<br>ᐞ *kindly fill form L*	Mean Gradient:<br>Peak gradient

**H5**	**PERIPARTUM DETAILS(tick applicable)**

H5.1 Date of delivery D D M M Y Y Y Y — Time of Delivery: H H : M M ○ AM ○ PM

**H5.2** Mode of delivery	Vaginal	Assisted vaginal	LSCS	
			Emergency	Elective
	○	○	○	○

H5.3 Type of Anaesthesia ○ Spinal ○ Epi Spinal ○ Epidural ○ GA

H5.4 Induction of labour done: ○ Yes ○ No Reason for induction:

H5.5 Indication for LSCS: ☐ Obstetric ____________________

☐ Cardiac ____________________

**H6**	**MATERNAL OUTCOME** (tick all applicable)

CARDIOVASCULAR OUTCOME

**H6.1** Heart failure		**H6.2** Cardiogenic shock	**H6.3** Pulmonary Embolism	**H6.4** CVA	**H6.5** Other thrombotic manifestations	**H6.6** Bleeding manifestations	**H6.7** Arrhythmia
Pulmonary edema	CHF						
☐	☐	☐	☐	☐	☐	☐	☐

H6.8 ☐ Others_______________

OBSTETRIC OUTCOME

**H6.9** PIH	**H6.10** Pre- eclampsia	**H6.11** Eclampsia	**H6.12**PPH	**H6.13** HELLP	**H6.14** CVT
☐	☐	☐	☐	☐	☐

H6.15 ☐ Others________________

**H6.16 Final outcome**	○ Death(details)	○ Discharge(details)

**H7**	**FETAL OUTCOME (tick applicable)**

**H7.1** Live Birth	**H7.2** Still born	**H7.3** IUD	**H7.4** IUGR	**H7.5** Abortion
○	○	○	○	○ Spontaneous ○ Induced

H7.6 If Live Birth:

Term	
Pre-term	

G7.8 APGAR score ☐

H7.7 Birth Weight Kgs ☐ . ☐☐☐

>4 kg	Large for gestational age	○
2.5-4 kg	Appropriate for gestational age	○
1.5-2.5 kg	Low birth weight	○
<1.5 kg	Very low birth weight	○

**H8**	**NEONATAL OUTCOME**

**H8.1** Neonatal death	**H8.2** Congenital anomalies	**H8.3** Embryopathy	**H8.4** Heart disease	**H8.5** Others_____________
☐	☐	☐	☐	☐

**H9**	**DISCHARGE DETAILS**

**H9.1** Functional Class at discharge (tick applicable)	Class I	Class II	Class III	Class IV
	○	○	○	○

Disability at the time of discharge ○ Yes ○ No Details_______________

H9.2 Mode of Contraception:
○ Nil ○ IUCD ○ OCP ○ Barrier method
○ Tubectomy ○ Others_______________

H9.3 Follow up date: D D M M Y Y Y Y

**H10**	Any other relevant information/ remarks:

SPECIFIC DISEASE PROFORMA

I. PROSTHETIC VALVE (FORM I)

I1 Pre-Valve replacement Interventions (if any): ☐ BMV ☐ CMC ☐ OTHERS

I2 Pre-operative Diagnosis:
Etiological diagnosis: ☐ RHD ☐ CHD ☐ DEGENERATIVE ☐ BCAV ☐ OTHERS________________
Pathological diagnosis: ☐ MR ☐ MS ☐ AR ☐ AS ☐ Others /Associated lesions____________

I3 Baseline prosthetic valve details

I3.1 Date of valve replacement:

I3.2 Done in institution:

**I3.3** Valve replaced *(tick the relevant)*	**I3.4** Valve size(mm)	**I3.5** Post op gradient (mmHg)	**I3.6** Type of valve *(tick the relevant)* Bio prosthetic	Single tilting disc	Bi- leaflet	Others Specify	**I3.7** Post op OAC	**I3.8** Post op INR	**I3.9** Post op Aspirin
Mitral ☐			○	○	○	○	Warfarin ○		○ Yes
Aortic ☐			○	○	○	○			
Tricuspid ☐			○	○	○	○	Acitrom ○		
Pulmonary ☐			○	○	○	○			○ No

I4 Pre-pregnancy complications *(if any):*

Bleeding	Thrombotic: PVT	CVA	Others	Others	
☐	☐	☐	☐	☐	INR during Complication________ Anti-coag during Complication________

I5 **ANTICOAGULANT THERAPY *(Please see back page)***

I6 **ECHOCARDIOGRAPHIC ASSESSMENT- F/U**

	**1st Tri**		**2nd Tri**		**3rd Tri**		**Peripartum**		**PN**	
	Mitral	Aortic	Mitral	Aortic	Mitral	Aortic	Mitral	Aortic	Mitral	Aortic
Peak gradient (mmHg)										
Peak velocity(m/s)										
Mean gradient (mmHg)										
Mean velocity(m/s)										
$VTI_{(prox)}/VTI_{(distal)}$										
EOA(cm2)										
PHT(ms)/ Aortic AT(ms)										
Normal 2D movement of the Prosthetic valve	○ Yes ○ No	○ Yes ○ No	○ Yes ○ No	○ Yes ○ No	○ Yes ○ No	○ Yes ○ No	○ Yes ○ No	○ Yes ○ No	○ Yes ○ No	○ Yes ○ No
Any thrombus visible	○ Yes ○ No	○ Yes ○ No	○ Yes ○ No	○ Yes ○ No	○ Yes ○ No	○ Yes ○ No	○ Yes ○ No	○ Yes ○ No	○ Yes ○ No	○ Yes ○ No

I7 **Present Pregnancy Complications**- Admitted ○ No ○ Yes. Duration of hospitalization___________________________

Prosthetic Valve Thrombosis
PVT ○ Yes ○ No
Time of PVT: ○ AN — Gestational age (in Weeks):
○ PN — Postnatal day:
Lysis done with: ○ Nil ○ SK ○ TNK
Outcome of Lysis : ○ Success ○ Failure
Anti-coagulant at the time of PVT
○ OAC ○ Heparin ○ UFH ○ LMWH
INR/APTT at time of PVT______________

Bleeding Complication
Bleeding: ○ Yes ○ No
Type of Bleed

Major	IC Bleed ☐	Retroperitoneal ☐	GIB ☐	Others ☐
Minor	Sub Cut ☐	Gum bleed ☐	Epistaxis ☐	Others ☐

Anti-coagulant at the time of Bleed
○ OAC ○ Heparin ○ UFH ○ LMWH
INR/APTT at time of Bleed______________

Other complications
☐ Other thrombotic complications
☐ Acute Pulmonary Edema
☐ Cardiogenic Shock
☐ Infective Endocarditis
☐ Others, Specify
____

I8 Any other relevant information/ remarks:

I 5. ANTICOAGULANT/ANTIPLATELET REGIMEN

Pre-pregnancy	○ ACITROM			○ WARFARIN Average INR_____			○ ASPIRIN														
Present pregnancy	1st trimester			2nd trimester			3rd trimester (upto 36wks)			Peripartum (>36 wks)			Intrapartum			Postpartum			Follow up		
ORAL ANTI-COAGULANTS	Dose	INR	Period	Dose	INR	Period	Dose	INR	Period	Dose	INR	Period	Dose	INR	Period	Dose	INR	Period	Dose		
ACITROM																					
WARFARIN																					
PARENTARAL ANTI-COAGULANTS																					
UFH	○ Infusion<br>○ Subcut<br>○ IV Bolus			○ Infusion<br>○ Subcut<br>○ IV Bolus			○ Infusion<br>○ Subcut<br>○ IV Bolus			○ Infusion<br>○ Subcut<br>○ IV Bolus			○ Infusion<br>○ Subcut<br>○ IV Bolus			○ Infusion<br>○ Subcut<br>○ IV Bolus			○ Infusion<br>○ Subcut<br>○ IV Bolus		
	Dose	APTT	Period	Dose	APTT	Period	Dose	APTT	Period	Dose	APTT	Period	Dose	APTT	Period	Dose	APTT	Period	Dose	APTT	Period
LMWH	Dose	ANTI XA	Period	Dose	ANTI XA	Period	Dose	ANTI XA	Period	Dose	ANTI XA	Period	Dose	ANTI XA	Period	Dose	ANTI XA	Period	Dose		
○ FONDAPARINUX<br>○ ENOXAPARIN<br>○ DALTEPARIN																					
ASPIRIN	○ No ○ Yes, Dose____			○ No ○ Yes, Dose____			○ No ○ Yes, Dose____			○ No ○ Yes, Dose____			○ No ○ Yes, Dose____			○ No ○ Yes Dose____			○ No ○ Yes Dose____		

J. CONGENITAL HEART DISEASES (FORM J)

J1 Disease specific *(tick the relevant)*

- ☐ ASD
- ☐ VSD
- ☐ PDA
- ☐ AVCD
- ☐ APW
- ☐ Other L→R shunts
- ☐ Tetralogy of Fallot (TOF)
- ☐ Ebsteins anomaly
- ☐ Transposition of Great Arteries-TGA
- ☐ Corrected transposition(cc-TGA)
- ☐ Double outlet right ventricle
- ☐ Pulmonary atresia
- ☐ Truncus arteriosus
- ☐ Single ventricle
- ☐ Tricuspid atresia
- ☐ Eisenmenger physiology (Also tick the relevant shunt above)
- ☐ Post surgical ☐ ICR ☐ Fontan ☐ BT shunt ☐ Others, specify__________
- ☐ Other Heart Disease, specify_____________

J2 Pre pregnancy Mx:

Phlebotomy ☐ Yes ☐ No

Post-surgical	Corrective Sx	Palliative Sx
DOS		

On medications *(if on medications tick the relevant)*

- ☐ Sildenafil
- ☐ Bosentan
- ☐ Vasodilators
- ☐ Iron supplements
- ☐ Others

J3 Pre-pregnancy complications:

☐ Cerebral abscess	☐ Bleeding manifestations	☐ Cardiac failure
☐ CVT	☐ Hemoptysis	☐ Others

J4 **Clinical findings during present pregnancy**

I4.1 Symptoms

- ☐ Headache
- ☐ Visual disturb
- ☐ Dizziness
- ☐ Altered mental status
- ☐ Bleeding tendency
- ☐ CNS symptoms
- ☐ Arthritis
- ☐ Renal dysfunction
- ☐ Cyanosis

J4.2 Polycythemia ○ Yes ○ No

J4.3 Anemia ○ Yes ○ No

J4.4 SPo2 (%)

J4.5 HCT(%) ________________

J4.6 S. ferritin __________________

J5 Echocardiographic assessment

Description:

Defect	Size	Location of the defect			
ASD		☐ Secondum	☐ Primum	☐ Sinus Venosus	☐ Others
VSD		☐ Perimembranous	☐ Outlet	☐ Inlet	☐ Others
PDA		Other echo description:			
APW					
AVCD					

J6 Any other relevant information/ remarks:

TNPHDR

K. CARDIAC FAILURE (FORM K)										
K1	Etiology of Cardiac failure *(tick the relevant)*									
	**K1.1** PPCMP	**K1.2** DCMP	**K1.3** IHD	**K1.4** VHD	**K1.5** HCMP	**K1.6** RCMP	**K1.7** MYOCARDITIS	**K1.8** CHD	**K1.9** ARF	**K1.10** NON-COMPACTION
	☐	☐	☐	☐	☐	☐	☐	☐	☐	☐
	**K1.11** ☐ Others (Specify):									
K2	Pre- pregnancy decompensation: ○ Yes No ○									
K3	Cardiac decompensation: ☐ Heart failure ☐ Acute pulmonary edema ☐ Cardiogenic shock					Treatment given: ☐ Hospitalization ☐ Ionotropic support ☐ Ventilatory support				
K4	**Clinical symptoms and signs during present pregnancy**									
	**K4.1** Fatigue/ Effort int	**K4.2** Ascites	**K4.3** Pleural effusion	**K4.4** Hepatomegaly	**K4.5** Cardiomegaly	**K4.6** Other				
	☐	☐	☐	☐	☐	☐				
K5	**PERIPARTUM CARDIOMYOPATHY**									
	Prior diagnosis of PPCMP *(If multigravida)* ○ Yes ○ No EF during diagnosis: EF before present pregnancy:									
	Present pregnancy	Symptoms at presentation:								
		Day when symptoms first noted: ○ AN (Gest. Age___________ wks) ○ PN: __________day								
		Antecedent history: ☐ Fever ☐ Obesity ☐ Family history ☐ Substance abuse								
		Treatment given:								
K6	Any other relevant information/ remarks:									

L. OUTFLOW TRACT OBSTRUCTIONS (FORM L)						
L1	AORTOPATHY AND LVOTO (tick the relevant)					
	**L1.1** Marfan	**L1.2** BCAV	**L1.3** Other Familial Syndromes	**L1.4** Aortic Dissection	**L1.5** Others	
	☐	☐	☐	○ Type A ○ Type B	☐	
L2	**Clinical symptoms and signs during present pregnancy**					
	**L2.1** Fatigue/ Effort int	**L2.2** Ascites	**L2.3** Pleural effusion	**L2.4** Hepatomegaly	**L2.5** Cardiomegaly	**L2.6** Others
	☐	☐	☐	☐	☐	☐

L3	**Echocardiographic assessment**						
	**LVOT OBSTRUCTION**						
	Aortic valve morphology ○Tricuspid ○Bicuspid ○Quadricuspid ○ Others						
	Site of LVOT Obstruction ☐ Valvar ☐ Supra Valvar ☐ Sub Valvar ○ Fixed ○ Dynamic						
	**PARAMETERS**		**1ST TRI**	**2ND TRI**	**3RD TRI**	**PERIPARTUM**	**PN**
	Aortic dimensions in mm	Annulus					
		Sinus					
		STJ					
		Asc. Aorta					
		Arch					
		Desc thoracic aorta					
	**AORTIC STENOSIS**						
	Gradient in mm of Hg	Mean					
		Peak					
	Velocity in m/sec	Mean					
		Peak					
	**RVOT Obstruction** Pulmonary valve morphology : ☐ Valvar ☐ Supra Valvar ☐ Sub Valvar						
	Gradient	Mean					
		Peak					
	Velocity	Mean					
		Peak					
L4	Any other relevant information/ remarks:						

FOLLOW UP VISIT PAGE-I

Date | D | D | M | M | Y | Y | Y | Y |

M1	**PATIENT SYMPTOMS**							
	**M1.1** NYHA CLASS ○ Class I ○ Class II ○ Class III ○ Class IV							
	Presenting symptoms (tick all applicable)							
	**M1.2** Nil	**M1.3** Dyspnea	**M1.4** PND	**M1.5** Orthopnea	**M1.6** Palpitation	**M1.7** Pedal edema	**M1.8** Chest pain	**M1.9** Syncope
	☐	☐	☐	☐	☐	☐	☐	☐
	**M1.10** ☐ **Others** *(Specify):*							

M2	**CLINICAL SIGNS & ECG**					
	**M2.1** Ht (cm)		**M2.2** Wt (Kg)		**M2.3** RR (/min)	
	**M2.4** HR (/min)		**M2.5** BP(mm Hg)		**M2.6** SPO2 (%)	
	**M2.7** CVS: S1______________ S2______________<br>Murmur: ○ Yes ○ No ☐ Sys ☐ Dias<br>Gallop: ○ Yes ○ No ☐ LVS3 ☐ RVS3		**L2.8** JVP<br>○ Normal<br>○ Elevated		**M2.9** RS ○ Normal ○ Abnormal<br>☐ Crepitations<br>☐ Rhonchi/ Wheeze	
	**M2.10 ECG** ○ Normal ○ Abnormal. If abnormal, Choose:<br>☐ AF ☐ SVT ☐ VT ☐ 1°AVB ☐ 2 AVB ☐ CHB ☐ ST↑ ☐ ST↓ ☐ **Others**<br>**Others, Specify:**					

M3	**ECHOCARDIOGRAPHIC ASSESSMENT**					
	**Ventricular function**				**Pulmonary pressures**	
	Wall Motion: ○ Normal ○ Global Hypokinesia<br>○ Regional wall motion abnormalities				Tricuspid regurgitation	TRPG (mmHg) :
					Pulmonary regurgitation	Peak PR (mmHg):
	LV systolic Function	LVID	Diastole(mm)		**Shunt lesion (Nature of shunt)**	○ L→R ○ R→L<br>○ Bi directional
			Systole(mm)			
		EF%			**Pericardial effusion** *(if present)*	○ Mild<br>○ Moderate<br>○ Massive
	LV diastolic function	E(cm/s)				
		A(cm/s)				
	RV systolic function	○ Normal ○ Abnormal<br>TAPSE (mm)__________ Sa'________			**Others**	☐ Vegetations ☐ Thrombus

Valve function (*For aortic & pulmonary stenosis kindly fill form L and prosthetic valve fill form* ***I***)

Mitral	☐ Normal	☐ Stenotic ○ Mild ○ Moderate ○ Severe<br>☐ Regurgitant ○ Mild ○ Moderate ○ Severe	MVOA:______________cm²<br>Mean Gradient:<br>Peak gradient:
Aortic	☐ Normal	☐ Stenotic$^{\Delta}$ ○ Mild ○ Moderate ○ Severe<br>☐ Regurgitant ○ Mild ○ Moderate ○ Severe	Mean Gradient:<br>Peak gradient :
Tricuspid	☐ Normal	☐ Stenotic ○ Mild ○ Moderate ○ Severe<br>☐ Regurgitant ○ Mild ○ Moderate ○ Severe	Mean Gradient:<br>Peak gradient:
Pulmonary	☐ Normal	☐ Stenotic$^{\Delta}$ ○ Mild ○ Moderate ○ Severe<br>☐ Regurgitant ○ Mild ○ Moderate ○ Severe	Mean Gradient:<br>Peak gradient:

M4	TNPHDR Risk category (as per triage): ○ LOW RISK ○ HIGH RISK
	On Medications:
M5	Hospitalisations after discharge: ○ Yes ○ No. If Yes, Reason__________________
M6	Post-partum plan
M7	**NEONATAL OUTCOME**
	**M7.1** Neonatal weight gain:
	**M7.2** Echocardiographic evaluation (if any)
	**M7.3** Adverse neonatal outcome: ○ Neonatal death ○ Cardiac lesion ○ Malnourishment
M8	Date of next follow up: D D M M Y Y Y Y

FOLLOW UP VISIT-II

Date D D M M Y Y Y Y

N1	**PATIENT SYMPTOMS**
	**N1.1** NYHA CLASS ○ Class I ○ Class II ○ Class III ○ Class IV
	Presenting symptoms (tick all applicable)

**N1.2** Nil	**N1.3** Dyspnea	**N1.4** PND	**N1.5** Orthopnea	**N1.6** Palpitation	**N1.7** Pedal edema	**N1.8** Chest pain	**N1.9** Syncope
☐	☐	☐	☐	☐	☐	☐	☐

N1.10 ☐ **Others** *(Specify):*

N2 CLINICAL SIGNS & ECG

**N2.1** Ht (cm)		**N2.2** Wt (Kg)		**N2.3** RR (/min)	
**N2.4** HR (/min)		**N2.5** BP(mm Hg)		**N2.6** SPO2 (%)	

**N2.7** CVS: S1______________ S2_______________<br>Murmur: ○ Yes ○ No ☐ Sys ☐ Dias<br>Gallop: ○ Yes ○ No ☐ LVS3 ☐ RVS3	**N2.8** JVP<br>○ Normal<br>○ Elevated	**N2.9** RS ○ Normal ○ Abnormal<br>☐ Crepitations<br>☐ Rhonchi/ Wheeze

N2.10 ECG ○ Normal ○ Abnormal. If abnormal, Choose:

☐ AF ☐ SVT ☐ VT ☐ 1°AVB ☐ 2 AVB ☐ CHB ☐ ST↑ ☐ ST↓ ☐ **Others**

Others, Specify:

N3 ECHOCARDIOGRAPHIC ASSESSMENT

**Ventricular function**				**Pulmonary pressures**	
Wall Motion: ○ Normal ○ Global Hypokinesia<br>○ Regional wall motion abnormalities				Tricuspid regurgitation	TRPG (mmHg) :
				Pulmonary regurgitation	Peak PR (mmHg):
LV systolic function	LVID	Diastole(mm)		**Shunt lesion (Nature of shunt)**	○ L→R ○ R→L<br>○ Bi directional
		Systole(mm)			
	EF%			**Pericardial effusion** *(if present)*	○ Mild<br>○ Moderate<br>○ Massive
LV diastolic function	E(cm/s)				
	A(cm/s)				
RV systolic function	○ Normal ○ Abnormal<br>TAPSE (mm)__________ Sa'_________			**Others**	☐ Vegetations ☐ Thrombus

Valve function (*For aortic & pulmonary stenosis kindly fill form L and prosthetic valve fill form I*)

Mitral	☐ Normal	☐ Stenotic ○ Mild ○ Moderate ○ Severe<br>☐ Regurgitant ○ Mild ○ Moderate ○ Severe	MVOA:_______________cm^2<br>Mean Gradient:<br>Peak gradient:
Aortic	☐ Normal	☐ Stenotic$^{\Delta}$ ○ Mild ○ Moderate ○ Severe<br>☐ Regurgitant ○ Mild ○ Moderate ○ Severe	Mean Gradient:<br>Peak gradient :
Tricuspid	☐ Normal	☐ Stenotic ○ Mild ○ Moderate ○ Severe<br>☐ Regurgitant ○ Mild ○ Moderate ○ Severe	Mean Gradient:<br>Peak gradient:
Pulmonary	☐ Normal	☐ Stenotic$^{\Delta}$ ○ Mild ○ Moderate ○ Severe<br>☐ Regurgitant ○ Mild ○ Moderate ○ Severe	Mean Gradient:<br>Peak gradient:

N4	TNPHDR Risk category (as per triage): ○ LOW RISK ○ HIGH RISK
	On Medications:
N5	Hospitalisations after discharge: ○ Yes ○ No. If Yes, Reason____________________
N6	Post-partum plan:
N7	**NEONATAL OUTCOME**
	**N7.1** Neonatal weight gain
	**N7.2** Echocardiographic evaluation (if any)
	**N7.3** Adverse neonatal outcome: ○ Neonatal death ○ Cardiac lesion ○ Malnourishment
N8	Date of next follow up: D D M M Y Y Y Y

TNPHDR

FOLLOW UP VISIT -III

Date | D | D | M | M | Y | Y | Y | Y |

O1	**PATIENT SYMPTOMS**
	**O1.1** NYHA CLASS ○ Class I ○ Class II ○ Class III ○ Class IV
	Presenting symptoms (tick all applicable)

**O1.2** Nil	**O1.3** Dyspnea	**O1.4** PND	**O1.5** Orthopnea	**O1.6** Palpitation	**O1.7** Pedal edema	**O1.8** Chest pain	**O1.9** Syncope
☐	☐	☐	☐	☐	☐	☐	☐

O1.10 ☐ **Others** *(Specify):*

O2 CLINICAL SIGNS & ECG

**O2.1** Ht (cm)		**O2.2** Wt (Kg)		**O2.3** RR (/min)	
**O2.4** HR (/min)		**O2.5** BP(mm Hg)		**O2.6** SPO2 (%)	

O2.7 CVS: S1______________ S2______________
Murmur: ○ Yes ○ No ☐ Sys ☐ Dias
Gallop: ○ Yes ○ No ☐ LVS3 ☐ RVS3

O2.8 JVP ○ Normal ○ Elevated

O2.9 RS ○ Normal ○ Abnormal ☐ Crepitations ☐ Rhonchi/ Wheeze

O2.10 ECG ○ Normal ○ Abnormal. If abnormal, Choose:
☐ AF ☐ SVT ☐ VT ☐ 1°AVB ☐ 2 AVB ☐ CHB ☐ ST↑ ☐ ST↓ ☐ **Others**

Others, Specify:

O3 ECHOCARDIOGRAPHIC ASSESSMENT

**Ventricular function**			**Pulmonary pressures**	
Wall Motion: ○ Normal ○ Global Hypokinesia ○ Regional wall motion abnormalities			Tricuspid regurgitation	TRPG (mmHg) :
			Pulmonary regurgitation	Peak PR (mmHg):
LV systolic function	LVID Diastole(mm)		**Shunt lesion (Nature of shunt)**	○ L→R ○ R→L ○ Bi directional
	LVID Systole(mm)			
	EF%		**Pericardial effusion** *(if present)*	○ Mild ○ Moderate ○ Massive
LV diastolic function	E(cm/s)			
	A(cm/s)			
RV systolic function	○ Normal ○ Abnormal TAPSE (mm)_________Sa'________		**Others**	☐ Vegetations ☐ Thrombus

Valve function (*For aortic & pulmonary stenosis kindly fill form L and prosthetic valve fill form I*)

Mitral	☐ Normal	☐ Stenotic ○ Mild ○ Moderate ○ Severe<br>☐ Regurgitant ○ Mild ○ Moderate ○ Severe	MVOA:______________cm²<br>Mean Gradient:<br>Peak gradient:
Aortic	☐ Normal	☐ Stenotic^Δ ○ Mild ○ Moderate ○ Severe<br>☐ Regurgitant ○ Mild ○ Moderate ○ Severe	Mean Gradient:<br>Peak gradient :
Tricuspid	☐ Normal	☐ Stenotic ○ Mild ○ Moderate ○ Severe<br>☐ Regurgitant ○ Mild ○ Moderate ○ Severe	Mean Gradient:<br>Peak gradient:
Pulmonary	☐ Normal	☐ Stenotic^Δ ○ Mild ○ Moderate ○ Severe<br>☐ Regurgitant ○ Mild ○ Moderate ○ Severe	Mean Gradient:<br>Peak gradient:

O4 TNPHDR Risk category (as per triage): ○ LOW RISK ○ HIGH RISK

On Medications:

O5 Hospitalisations after discharge: ○ Yes ○ No. If Yes, Reason___________________

O6 Post-partum plan

O7 Date of next follow up: | D | D | M | M | Y | Y | Y | Y |
