# Supplementary material for: Tamil Nadu Pregnancy and Heart Disease Registry (TNPHDR): design and methodology
Source: BMC Pregnancy Childbirth. 2022 Jan 29;22:80. doi: 10.1186/s12884-021-04305-3 (PMC8801092; doi:10.1186/s12884-021-04305-3)
Supplement: Supplementary file 3 — Additional file 3: Standard operating procedure [file 12884_2021_4305_MOESM3_ESM.doc]

# TAMIL NADU PREGNANCY

# AND HEART DIESEASE REGISTRY

**Funded by Indian Council of Medical Research**

**STANDARD OPERATING PROCEDURE – Enrolment & CRF filling**

**Study Process**

All antenatal women seeking outpatient or inpatient care in any of the participating sites, with known or newly diagnosed structural heart disease, cardiac rhythm disorders, aortopathy or aortic vascular diseases are eligible for inclusion in the study. Antenatal patients can be enrolled at any trimester when they first enter into the institution. Women with heart disease seen for the first time after delivery can be included up to six weeks postpartum. Inclusion for women with peripartum cardiomyopathy is allowed up to six months postpartum.

Patients with pregnancy related complications like preexisting or gestational hypertension, anemia, eclampsia, gestational diabetes without structural heart disease were not included. The study enrollment period will be from 15th of January 2020 to 31st March 2021 or till 2500 participants are enrolled**.**

Patient will be explained in detail about the registry in vernacular and a detailed informed consent will be obtained before enrollment. Each enrolled patient will be assigned a unique autogenerated TNPHDR identification number by which they will be identified subsequently. Once enrolled, antenatal mothers and their families will be counselled about their cardiac condition, its impact on maternal and fetal outcome, the importance of periodic monitoring and pharmacotherapy and the role of healthcare in supporting them through the pregnancy.

**Duration and follow up**

Phase 1 of the study will continue up to March 2021 or till 2500 patients are enrolled. Phase II of the study will continue for 6 months after delivery of the last enrolled TNPHDR patient.

The patients will be followed up for at least 6 months after delivery / termination of pregnancy. During this period, data will be collected at 1 month, 3 month and 6 months’ time points. Follow up will be done during clinical visit or through telephonic calls. However, at least one of the follow up needs to be clinical, including an echocardiographic evaluation.

**Filling of Case Report Form (CRF):**

The case report form booklets (Supplement B) have been distributed to the Principal investigators of the various sites. Training to fill up the offline and online form has been given. The following Table 1 gives the explanation and support for details of the data to be collected under various heads / subheads in the CRF. The state coordinating center is available for help in filling up the form.

State coordination center can be approached for help in filling the form by telephone numbers (044 29550097) or email [tnphdr2019@gmail.com](mailto:tnphdr2019@gmail.com). In addition, the online CRF form provides help link, through which the investigators can reach out to the state coordinating center. The state coordinating center will periodically review the data uploaded from the sites for completeness and appropriateness and partner with the sites in improving the validity of the data uploaded.

| **Table I: Explanation for Case Report Form** | | | | |
| --- | --- | --- | --- | --- |
|  | | Data collected | Description | |
| A1 | | TNPHDR number | Auto generated 12 digit numbers. Patient ID has 3 parts. First four digits representing the year of registration/next three digits are the alphabetical code assigned to the sites/last five digits are the patient identification number | |
| A2 | | RCH number | 12 digit PICME (Pregnancy and Infant Cohort Monitoring and Evaluation) number | |
| A3 | | Date of Registration | Date when the antenatal/postnatal mother is first seen in the participating site | |
| **INCLUSION CRITERIA CHECKLIST** | | | | |
| A4 | | Period of enrollment | Enrolled as antenatal/ post-partum(upto 6 weeks)/ post natal(upto 6 months) | |
| A5 | | Criteria | This includes patients with structural heart disease, rhythm disturbances, systemic and pulmonary vascular diseases | |
| A 6 | | Consent | Consent in vernacular language | |
| **B. BASELINE DATA-IDENTIFIER PAGE** | | | | |
| B1 | Hospital/PIN number | | Enter correct hospital number for future reference | |
| B2 | Full name of the patient | | Write patient’s full name in CAPITAL letters | |
| B3 | Name of the Husband/Guardian | | Enter the name of the husband/ Guardian | |
| B4 | Age | | In completed years | |
| B5 | Date of birth | | day-month-year format | |
| B6 | House/Flat name or number | | Enter house/Flat name or number | |
| B7 | Street/locality | | Enter street/locality | |
| B8 | State | | Name of the State/Union territory where the patient is living | |
| B9 | District | | Name of the district where the patient is staying | |
| B10 | Taluk | | Enter taluk name | |
| B11 | Village | | Enter village name | |
| B12 | Post office | | Name of the post office | |
| B13 | Pin code | | Enter pin code | |
| B14 | Adhaar number | | Enter correctAadhaar number | |
| B15 | Patient’s mobile number | | Must be correct with 10 digits | |
| B16 | Patient’s alternate mobile number | | Patient second contact number | |
| B17 | Relative's mobile number | | Patient’s close relative’s phone number | |
| B18 | Total no of years of education | | Enter total number of years of education, qualification of the patient | |
| B19 | Occupation | | Enter occupation | |
| B20 | Marital status | | Enter marital status | |
| B21 | Total monthly income | | Total monthly income of the family | |
| B22 | Total members in the family | | Number of members in the family | |
| B23 | Socioeconomic status | | Enter socioeconomic status as per ration card as above or below poverty line | |
| **C. REGISTRATION DETAILS-REGISTRATION PAGE** | | | | |
|  | | **Time of first diagnosis of heart disease** |  | |
| C1 | | Age of diagnosis | Enter chronological age at diagnosis in years which is required in all patients including those diagnosed during pregnancy.  Gestational age at diagnosis is to be filled, if the heart disease was detected either during the present pregnancy or during a past pregnancy at diagnosis. Fill the gestational age in weeks when a patient was diagnosed during pregnancy.  Include data on whether diagnosed was made before or after marriage; during previous pregnancy or present pregnancy | |
|  | | **Pre-pregnancy details** | | |
| C2 | | Pre-pregnancy cardiac and related history | | |
| C2.1 | | NYHA class | New York Heart Association functional classification(explained below) | |
| C2.2 | | Prior HF | Earlier medical report showing diagnosis or treatment for heart failure or a definite clear history from patient. | |
| C2.3 | | Prior AF | Earlier medical report showing diagnosis or treatment of atrial fibrillation. An irregular, rapid heart rate that may cause symptoms like heart palpitations, fatigue, and shortness of breath. | |
| C2.4 | | Prior procedures | Enter details of any cardiac interventional procedures or cardiac surgeries that have been done in the past for congenital or acquired heart diseases both surgical and catheter based including balloon valvotomies | |
| C2.5 | | Prior OAC use | Includes acitrom (acenocoumaral), warfarin and newer drugs such as apixaban, dabigatran, rivaroxaban, and edoxaban | |
| C2.6 | | Others | Other relevant pre-pregnancy cardiac details | |
| C2.7 | | Details of prior procedures done (Surgical procedures or Cardiac interventions) | 1. Mitral: Closed mitral commissurotomy, Open mitral commissurotomy, Mitral valve replacement, Mitral annuloplasty 2. Tricuspid: Tricuspid valve replacement, Tricuspid annuloplasty 3. Aortic: Balloon aortic valvotomy, Aortic valve replacement 4. Pulmonary: Balloon pulmonary valvotomy, pulmonary valve replacement 5. Device/patch closure: ASD, VSD, PDA (others: RSOV) 6. Intracardiac repair 7. Fontan’s surgery 8. Arterial switch surgery 9. BT shunts and other palliative surgeries 10. Bentall surgery 11. Coarctation repair/ stenting 12. Coils, IVC filters | |
| C2.8 | | Prior Hospitalizations | Due to cardiac causes/complication such as pulmonary edema, cardiogenic shock, arrhythmias, infective endocarditis, OAC associated bleeding/thrombosis | |
| C2.9 | | Medications | Mention all the medications patient is on | |
| C2.10 | | Diabetes | Mention diabetic status | |
| C2.11 | | SHT | Mention history of hypertension | |
| C2.12 | | CAD | Mention history of Coronary Artery disease | |
| C2.13 | | Alcohol | Mention history of alcohol consumption | |
| C2.14 | | Smoking | Mention history of smoking | |
| C2.15 | | Tobacco use | Mention history Tobacco use | |
| C2.16 | | Others | Enter any other relevant history that needs mention | |
| C3 | | **Previous pregnancy details***(Kindly add additional page for more than one previous pregnancy)* | | |
| C3.1 | | Date of delivery/termination of previous pregnancy | Enter the Date of delivery/termination of previous pregnancy | |
| C3.2 | | Gestational age at delivery | Enter the Gestational age at delivery in weeks | |
| C3.3 | | Mode of delivery | Enter the mode of previous delivery: LSCS/ vaginal/ assisted vaginal | |
| C3.4 | | Foetal outcome | Mention the foetal outcome: Live birth, preterm, still birth/ IUD/ abortion(induced or spontaneous)/ low birth weight/ small for age(explained below) | |
| C3.5 | | AN/PN decompensation | Enter any antenatal/ postnatal decompensation during previous pregnancy: Heart failure/ cardiogenic shock/ bleeding or thrombotic complication / arrhythmia/ infective complication/ mention any other complications | |
| C3.6 | | Any antenatal interventions done | Any antenatal interventions done in previous pregnancy, include the name of the procedure, institution where done and gestational age during procedure | |
| C3.7 | | Post-partum interventions | Post-partum interventions in previous pregnancy include the name of the procedure, institution where done and postnatal day during procedure | |
| C3.8 | | Was the previous pregnancy continued Against Medical Advice | Mention if the previous pregnancy continued Against Medical Advice | |
| **Present pregnancy details** | | | | |
| C4 | | Obstetric score | Mention the Obstetric score and also whether singleton or twin pregnancy or others | |
| C5 | | LMP | Last Menstrual Period | |
| C6 | | EDD | Expected Date of Delivery | |
| C7 | | Gestational age | Gestational age at registration in TNPHDR (in weeks) | |
| C8 | | Antenatal check-ups | | |
| C8. 1 | | Booked / Unbooked / Status not known | | A pregnant woman is said to have “booked” if she attended at least three antenatal clinic visits and received at least one dose of tetanus immunization. She is also considered “ booked” if, she makes a minimum of two more visits lasting not more than two weeks before delivery. A pregnant women who have no prenatal care at all throughout the pregnancy or had less than two antenatal clinic visits  Mention whether booked/ unbooked or status not known |
| C8. 2 | | Institution where AN check-up done | | Enter institution where patient is having her AN check-up done. This may include government primary health centres, taluk hospitals, head quarters hospitals, medical colleges and private institutions |
| C9 | | **Associated risk factor** | | |
| C9.1 | | PIH/GHTN | | Pregnancy Induced Hypertension/ Gestational Hypertension  Gestational hypertension is defined as a systolic blood pressure 140 mm Hg or more or a diastolic blood pressure of 90 mm Hg or more, or both, on two occasions at least 4 hours apart after 20 weeks of gestation, in a woman with a previously normal blood pressure |
| C9.2 | | GDM | | Gestational Diabetes Mellitus  GDM is diagnosed if at least two of the following four plasma glucose levels (measured during OGTT) are met or exceeded Fasting: 95 g/dL (5.3 mmol/L), 1 hour: 180 mg/dL (10.0 mmol/L), 2 hour: 155 mg/dL (8.6 mmol/L), 3 hour: 140 mg/dL (7.8 mmol/L) |
| C9.3 | | Hypothyroid | | Mention if Hypothyroid: TSH levels more than the upper reference limit of 2.5 mU/L in the first trimester and 3.0 mU/ L in the second and third trimesters |
| C9.4 | | Hyperthyroid | | Mention if Hyperthyroid: TSH levels less than the lower reference limit of below 0.1 mU/L |
| C9.5 | | Anaemia | | Mention if anemic: Hemoglobin levels < 11 g/dl |
| C9.6 | | Rheumatologic problems | | Mention any Rheumatologic problems: : *Rheumatic diseases* are characterized by inflammation that affects the connecting or supporting structures of the body — most commonly the joints, but also sometimes the tendons, ligaments, bones, and muscles. ([Rheumatoid arthritis](https://www.webmd.com/rheumatoid-arthritis/default.htm) (RA),[Lupus](https://www.webmd.com/lupus/default.htm), Spondyloarthropathies -- [ankylosing spondylitis](https://www.webmd.com/back-pain/guide/ankylosing-spondylitis) (AS) Sjogren’s syndrome, [Gout](https://www.webmd.com/arthritis/arthritis-gout), [Scleroderma](https://www.webmd.com/pain-management/scleroderma), [Infectious arthritis](https://www.webmd.com/arthritis/septic-arthritis-symptoms-diagnosis-and-treatment), [Juvenile idiopathic arthritis](https://www.webmd.com/rheumatoid-arthritis/tc/juvenile-rheumatoid-arthritis-what-happens), [Polymyalgia rheumatica](https://www.webmd.com/arthritis/polymyalgia-rheumatica-temporal-arteritis) |
| C9.7 | | Others | | Mention any other relevant associated risk factors |
| C10 | | **COMPLETE DIAGNOSIS** | | Mention the complete diagnosis. This should include anatomical, etiological and pathological diagnosis |
| C11 | | Present pregnancy continued Against Medical Advice | | Mention if the present pregnancy continued Against Medical Advice. Patient continuing her pregnancy despite multidisciplinary counselling with complete knowledge of the maternal risks |
| C12 | | Any antenatal interventions done before registration | | Mention if any antenatal interventions done before registration |
| C13 | | Post-partum interventions | | Only for cases registered in TNPHDR within 6weeks of delivery |
| **D. FIRST VISIT PAGE** | | | | |
| D1 | | **Patient symptoms** | | |
| D1.1 | | NYHA | | Class I - No symptoms and no limitation in ordinary physical activity.  Class II - Mild symptoms and slight limitation during ordinary activity.  Class III - Marked limitation due to symptoms, during less-than-ordinary activity, e.g. walking short distances (20—100 m). Comfortable only at rest.  Class IV - Severe limitations with symptoms at rest. Symptoms: Shortness of breath or dyspnea / angina/ palpitation |
| D1.2 | | Nil | | Tick if the patient is asymptomatic |
| D1.3 | | Dyspnea | | Difficult or Labored breathing |
| D1.4 | | PND | | Severe shortness of breath and coughing that generally occur at night. It usually awakens the person from sleep, and may be quite frightening |
| D1.5 | | Orthopnea | | Shortness of breath (dyspnea) that occurs when lying flat |
| D1.6 | | Palpitation | | Feeling of having a fast-beating, fluttering or pounding heart |
| D1.7 | | Pedal edema | | Accumulation of fluid in the feet and lower legs |
| D1.8 | | Chest pain | | Mention any compressive chest pain. If atypical kindly mention |
| D1.9 | | Syncope | | Transient loss of consciousness with an inability to maintain postural tone that is followed by spontaneous recovery |
| D1.10 | | Others | | Mention any other symptoms the patient has |
| D2 | | **Clinical signs** | | |
| D2.1 | | Height | | Mention patient’s height in cms |
| D2.2 | | weight | | Mention patients’ weight in Kgs |
| D2.3 | | Respiratory rate | | Respiratory rate per minute |
| D2.4 | | Heart rate | | Heart rate per minute |
| D2.5 | | Blood pressure | | Blood pressure measured in mmHg |
| D2.6 | | SPO2 | | Arterial oxygen saturation evaluated with pulse oximeter |
| D2.7 | | CVS | | Cardiovascular system examination for heart sounds and murmurs |
| D2.8 | | JVP | | Jugular Venous Pressure: normal/ elevated |
| D2.9 | | RS | | Respiratory system evaluation for sounds/ wheeze/ crepitations |
| D3 | | **Baseline investigations** | | |
| D3.1 | | Blood Sugar | | Blood Sugar (mg/dl) |
| D3.2 | | Blood Urea | | Blood Urea (mg/dl) |
| D3.3 | | Sr. Creatinine | | Sr. Creatinine (mg/dl) |
| D3.4 | | Hb | | Hemoglobin in g/dl |
| D3.5 | | Others | | Mention any others relevant investigations |
| D3.6 | | ECG | | Abnormal ECGs  AF- Atrial Fibrillation  SVT- Supra Ventricular Tachycardia  VT - Ventricular Tachycardia  1°AVB - First degree Atrio-ventricular Block  2ᴼAVB - Second degree Atrio-ventricular Block  CHB - Complete Heart Blok  ST↑ - ST segment elevation  ST↓ - ST segment depression |
| **D4** | | **RISK ASSESSMENT AND TRIAGE** | | |
| D4.1 | | Baseline risk | | Mention mWHO risk class See *Table -II* |
| D4.2 | | CARPREG I SCORE | | *See Table -III* |
| D4.3 | | CARPREG II SCORE | | *See Table -III* |
| D4.4 | | ZAHARA SCORE | | *See Table -III* |
| **D5** | | ECHOCARDIOGRAPHIC ASSESSMENT | | **Detailed echocardiographic evaluation for** Situs, VA concordance, AV concordance, Chamber description, Ventricular function, Valve morphology and function Pulmonary pressures, Septal intactness, shunts, pericardium, aorta and other important findings |
| D6 | | TNPHDR Risk category | | See *Table -IV* |
| D7 | | Specific Advice | | Specify advice given during the visit like anticoagulant management and heparin switch overs, drugs titration, anti-failure drugs and admission advised. |
| D8 | | Date of next follow up | | Mention the date when the patient is supposed to come for next visit |
| **E. DRUG PAGE-USE AND DOSAGE** *(Table-V)* | | | | |
| E1 | | Pre- pregnancy | | Tick the drugs taken pre pregnancy |
| E2 | | I trimester | | Tick the drugs taken during I trimester |
| E3 | | II trimester | | Tick the drugs taken during II trimester |
| E4 | | III trimester | | Tick the drugs taken during III trimester |
| E5 | | Peripartum | | Tick the drugs taken during Peripartum |
| **F. SECOND VISIT PAGE** | | | | |
| F1 | | Patient symptoms | | Mention NYHA class and specify if patient is asymptomatic or if patient has Dyspnea, PND, Orthopnea, Palpitation, Pedal edema, Chest pain, Syncope, Others |
| F2 | | Clinical signs and ECG | | Mention height, weight, Respiratory rate, Heart rate, Blood pressure, SPO2, CVS, JVP, RS, ECG |
| F3 | | Echocardiographic assessment | | Details of focused echocardiogram to be mentioned |
| F4 | | Specific Advice in the visit | | Specify advice given during the visit like anticoagulant management, drugs titration, anti-failure drugs and admission advised. |
| F5 | | TNPHDR Risk category | | *See Table IV* |
| F6 | | Date of next follow up | | Mention the date when the patient is supposed to come for next visit |
| **G. THIRD VISIT PAGE** | | | | |
| G1 | | Patient symptoms | | Mention NYHA class and specify if patient is asymptomatic or if patient has Dyspnea, PND, Orthopnea, Palpitation, Pedal edema, Chest pain, Syncope, Others |
| G2 | | Clinical signs and ECG | | Mention height, weight, Respiratory rate, Heart rate, Blood pressure, SPO2, CVS, JVP, RS, ECG |
| G3 | | Echocardiographic assessment | | Details of focused echocardiogram to be mentioned |
| G4 | | Specific Advice in the visit | | Specify advice given during the visit like anticoagulant management, drugs titration, anti-failure drugs and admission advised. |
| G5 | | TNPHDR Risk category | | *See Table IV* |
| G6 | | Date of next follow up | | Mention the date when the patient is supposed to come for next visit |
| **H. PERIPARTUM VISIT PAGE** | | | | |
| H1 | | Patient symptoms | | Mention NYHA class and specify if patient is asymptomatic or if patient has Dyspnea, PND, Orthopnea, Palpitation, Pedal edema, Chest pain, Syncope, Others |
| H2 | | RISK ASSESSMENT AND TRIAGE | | |
| H2.1 | | Baseline risk | | Mention the mWHO class |
| H2.2 | | TNPHDR Risk category | | Mention the TNPHDR risk category |
| H3 | | Clinical signs and ECG | | Mention height, weight, Respiratory rate, Heart rate, Blood pressure, SPO2, CVS, JVP, RS, ECG |
| H4 | | Echocardiographic assessment | | Details of focused echocardiogram to be mentioned |
| H5 | | **PERIPARTUM DETAILS** | | |
| H5.1 | | Date of delivery | | Mention the correct date and time of delivery |
| H5.2 | | Mode of delivery | | Mention if LSCS/ vaginal or assisted vaginal |
| H5.3 | | Type of Anesthesia | | Mention if anesthesia given is spinal/epidural/epispinal / GA |
| H5.4 | | Induction of labor done | | Mention if induced and reason for induction |
| H5.5 | | Indication for LSCS | | Mention if obstetric or cardiac indication for LSCS |
| H6 | | **MATERNAL OUTCOME** | | |
|  | | **Cardio-vascular outcome** | | |
| H6.1 | | Heart failure | | Heart Failure was defined as presentation with symptoms of HF characterized by breathlessness, ankle swelling and fatigue with elevated jugular venous pressure, pulmonary crackles and peripheral edema) requiring therapy. Heart failure is considered as an event when it necessitated a new therapy or escalation of therapy or hospitalization |
| H6.2 | | Cardiogenic shock | | Cardiogenic shock is defined as a sustained reduction is systolic pressure of <90 mmHg caused by a cardiac index of less than 2.2 liters/min/mm^2^ |
| H6.3 | | Pulmonary Embolism | | Acute pulmonary embolism is defined as sustained hypotension (systolic blood pressure <90 mm Hg for at least 15 minutes or requiring inotropic support, not due to a cause other than PE, such as arrhythmia, hypovolemia, sepsis, or left ventricular [LV] dysfunction), pulselessness, or persistent profound bradycardia (heart rate <40 bpm with signs or symptoms of shock) |
| H6.4 | | CVA | | Cerebrovascular accident |
| H6.5 | | Other thrombotic manifestations | | Thromboembolic events include thrombotic or embolic events in both systemic and pulmonary circulation which could be a stroke or transient ischemic attack of cardiac origin, prosthetic valve thrombosis, and pulmonary embolism. |
| H6.6 | | Bleeding manifestations | | Hemorrhagic complications included both major (intracranial bleed, retroperitoneal hematoma), minor bleed (superficial skin bleed, epistaxis) and postpartum haemorrhage. |
| H6.7 | | Arrhythmia | | Symptomatic tachyarrhythmia, defined as abnormal heart rhythms with a ventricular rate of 100 or more beats per minute with symptoms of palpitations, diaphoresis, dyspnea, chest pain, dizziness, syncope, or heart failure. Symptomatic bradyarrhythmia is defined as ventricular rates less than 60 beats per minute directly responsible for development of the clinical manifestations of syncope or presyncope, transient dizziness or lightheadedness, heart failure symptoms, or confusional states resulting from cerebral hypoperfusion attributable to slow heart rate |
| H6.8 | | Others | | Mention any other cardiac event |
|  | | **Obstetric outcome** | | |
| H6.9 | | PIH/GHTN | | Pregnancy Induced Hypertension/ Gestational Hypertension  Gestational hypertension is defined as a systolic blood pressure 140 mm Hg or more or a diastolic blood pressure of 90 mm Hg or more, or both, on two occasions at least 4 hours apart after 20 weeks of gestation, in a woman with a previously normal blood pressure |
| H6.10 | | Pre- eclampsia | | Preeclampsia is a disorder of pregnancy associated with new-onset hypertension, which occurs most often after 20 weeks of gestation and frequently near term. |
| H6.11 | | Eclampsia | | The convulsive manifestation of the hypertensive disorders of pregnancy and is among the more severe manifestations of the disease. Eclampsia is defined by new-onset tonic-clonic, focal, or multifocal seizures in the absence of other causative conditions such as epilepsy, cerebral arterial ischemia and infarction, intracranial hemorrhage, or drug use |
| H6.12 | | PPH (Postpartum hemorrhage) | | Blood loss of more than 500 mL following vaginal delivery or more than 1000 mL following cesarean delivery. |
| H6.13 | | HELLP | | Hemolysis, Elevated Liver enzyme levels, and Low Platelet levels with increased LDH (> 600 U/L), AST (≥ 70 U/L), and platelets < 100·10^9^/L |
| H6.14 | | CVT (Cerebral Venous sinus Thrombosis) | | Headache, seizures, altered consciousness, and neurological focal signs on physical examination proven by neuro imaging |
| H6.15 | | Others | | Mention any other complications |
| H6.16 | | Final outcome | | Mention whether death or discharge  Maternal death was defined as death of the mother during pregnancy or within 7 days post-partum  Mention a detailed report of death and discharge whichever applicable |
| H7 | | **FETAL OUTCOME** | | |
| H7.1 | | Live Birth | | A live birth is the complete expulsion or extraction from its mother of a product of conception, irrespective of the duration of pregnancy, which, after such separation, breathes or shows any other evidence of life, such as beating of the heart, pulsation of the umbilical cord, or any definite movement of voluntary muscles, whether or not the umbilical cord has been cut or the placenta is attached. |
| H7.2 | | Still born | | Death in utero at or after 24 weeks of  pregnancy |
| H7.3 | | IUD | | Intrauterine death |
| H7.4 | | IUGR | | Small-for-gestational-age / Low birth weight  Birth weight <10th percentile for gestational age or less than 2.5 kg |
| H7.5 | | Abortion | | Miscarriage or abortion is termination or pregnancy prior to 24 weeks of gestation or a fetus born weighing less than 500 g |
| H7.6 | | Term/Pre-term | | Premature birth is birth before 37 weeks of gestation |
| H7.7 | | Birth Weight | | Mention the birth weight in Kgs |
| H7.8 | | APGAR score | | Mention the APGAR score |
| H8 | | **NEONATAL OUTCOME** | | |
| H8.1 | | Neonatal death | | Deaths occurring within 7 days of birth were early neonatal deaths |
| H8.2 | | Congenital anomalies | | Any form of congenital anomalies in the new born |
| H8.3 | | Embryopathy | | A developmental abnormality of an embryo or fetus especially when caused by maternal disease or drug exposure or infection. |
| H8.4 | | Heart disease | | Congenital heart disease noted in the new bon |
| H8.5 | | Others | | Any other relevant details pertaining to the new born |
| H9 | | **DISCHARGE DETAILS** | | |
| H9.1 | | Functional Class at discharge | | Mention the NYHA class at time of discharge |
| H9.2 | | Mode of Contraception | | Mention any contraception given or not: IUCD/ OCP/ tubectomy |
| H9.3 | | Date of next follow up | | Mention the date when the patient is supposed to come for next visit |
| H10 | | Other relevant information/remarks | | Mention any other relevant information |
| **SPECIFIC DISEASE PROFORMA** | | | | |
| I**. PROSTHETIC VALVE (FORM** I**)** | | | | |
| I1 | | Pre-Valve replacement Interventions | | Mention any Pre-Valve replacement Interventions like Balloon mitral valvotomy(BMV), Closed mitral commissurotomy(CMC) or any other valvular surgery |
| I2 | | Pre-operative Diagnosis | | Mention the complete pre-operative diagnosis:  Etiological diagnosis: Rheumatic Heart Disease(RHD), Congenital heart disease(CHD),Degenerative valve disease, Bicuspid Aortic Valve( BCAV) and others if any  Pathological diagnosis:  MR- Mitral Regurgitation  MS- Mitral Stenosis  AR- Aortic Regurgitation  AS- Aortic Stenosis  Others lesions: Tricuspid and pulmonic valve stenosis/ regurgitations |
| I3 | | Baseline prosthetic valve details | | Mention baseline prosthetic details  Date of valve replacement, Institution where done, valve which was replaced, valve size, type of valve  Post-operative details of gradient, oral anticoagulants(OAC), antiplatelet, INR |
| I4 | | Pre-pregnancy complications | | Mention any pre pregnancy prosthetic valve related complications: bleeding or thrombotic event, Anticoagulant and INR during that episode |
| I5 | | Anticoagulant therapy *(Table -VI)* | | |
| I6 | | Echocardiographic assessment | | Details prosthetic valve echocardiographic parameters |
| I7 | | Present Pregnancy Complications | | Mention any present pregnancy events and duration of hospitalization  Prosthetic valve thrombosis: Time of the event, whether lysis done, outcome, anticoagulant during prosthetic thrombosis  Bleeding event: Major bleed(intracranial bleed, retroperitoneal bleed, Gastrointestinal bleed), minor bleed(epistaxis, gum bleed, subcutaneous bleed), anticoagulant during bleeding episode  Other complications: pulmonary edema, cardiogenic shock, infective endocarditis |
| I8 | | Any other relevant information | | Mention any other relevant valve related information/events |
| **J. CONGENITAL HEART DISEASES (FORM J)** | | | | |
| J1 | | Disease specific | | *Tick the relevant congenital heart disease (Acyanotic and cyanotic heart disease from the list provided*  ASD – Atrial Septal Defect  VSD- Ventricular Septal Defect  PDA- Patent Ductus Arteriosus  AVCD- AV canal defect  APW- AP window  Other LàR shunts  TOF- Tetralogy of Fallot  Ebstein’s anomaly  Transposition of Great Arteries-TGA  Corrected transposition(cc-TGA)  Pulmonary atresia  Truncus arteriosus  Single ventricle  Tricuspid atresia |
| J2 | | Pre pregnancy management | | Mention any prior phlebotomy, prior palliative or corrective surgery done, medications the patient is presently on |
| J3 | | Pre- pregnancy complications | | Mention any pre pregnancy complications: Cerebral abscess, Cardiac failure, CVT, Hemoptysis Other bleeding manifestations, Others |
| J4 | | Clinical findings during present pregnancy | | **Symptoms:** Bleeding tendency, Polycythemia, Headache, Visual disturb, Dizziness, Altered mental status,CNS symptoms, Anemia  Arthritis  **Signs** SPo2 (%),HCT(% Renal dysfunction ,Cyanosis S. ferritin |
| J5 | | Echocardiographic assessment | | Detailed description of the congenital heart disease |
| J6 | | Any other relevant information | | Mention any other relevant valve related information/events |
| **K. CARDIAC FAILURE (FORM K)** | | | | |
| K1 | | Etiology of Cardiac failure | | PPCMP- Peripartum cardiomyopathy  DCMP- Dilated cardiomyopathy  IHD- Ischemic heart disease  VHD- Valvular Heart disease  HCMP- Hypertrophic cardiomyopathy  RCMP - Restrictive cardiomyopathy  MYOCARDITIS  CHD- Congenital Heart Disease  ARF-Acute Rheumatic Fever  NON-COMPACTION- Non compaction cardiomyopathy |
| K2 | | Pre- pregnancy decompensation | |  |
| K3 | | Cardiac decompensation | | Any forms of cardiac failure, pulmonary edema, cardiogenic shock and treatment given(whether on ventilator or ionotrophic support) |
| K4 | | Clinical symptoms and signs during present pregnancy | | Mention any signs of heart failure: Fatigue, effort intolerance/ ascites/hepatomegaly/cardiomegaly |
| K5 | | **PERIPARTUM CARDIOMYOPATHY** | | If diagnosed during previous pregnancy mention if Ejection Fraction (EF) has recovered.  Present pregnancy: Mention symptoms of presentation, day of symptom, antecedent history(fever, PIH, family history etc) and treatment given |
| K6 | | Any other relevant information | | Mention any other relevant information/events |
| **L. OUTFLOW TRACT OBSTRUCTIONS (FORM L)** | | | | |
| L1 | | AORTOPATHY AND LVOTO | | Mention any cause of aortopathy like marfans, BCAV or other familial syndromes |
| L2 | | Clinical symptoms and signs during present pregnancy | | Mention any symptoms: Fatigue, effort intolerance/ ascites/ hepatomegaly/ cardiomegaly |
| L3 | | Echocardiographic assessment | | Details of focused echocardiogram |
| L4 | | Any other relevant information | | Mention any other relevant information/events |
| **M. FOLLOW UP VISIT -1** | | | | |
| M 1 | | Patient symptoms | | Mention NYHA class and specify if patient is asymptomatic or if patient has Dyspnea, PND, Orthopnea, Palpitation, Pedal edema, Chest pain, Syncope,Others |
| M 2 | | Clinical signs and ECG | | Mention height, weight, Respiratory rate, Heart rate, Blood pressure, SPO2, CVS, JVP, RS, ECG |
| M 3 | | Echocardiographic assessment | | Details of focused echocardiogram to be mentioned |
| M 4 | | TNPHDR Risk category | | Mention TNPHDR Risk category |
| M 5 | | Hospitalisations after discharge | | Mention any hospitalization after discharge |
| M 6 | | Post-partum plan | | Mention the postpartum treatment details and any interventions if suggested |
| M 7 | | **NEONATAL OUTCOME** | | Mention the neonatal weight gain, any echo evaluation done any adverse events |
| M 8 | | Date of next follow up: | | Mention the date when the patient is supposed to come for next visit |
| **N. FOLLOW UP VISIT -2** | | | | |
| N 1 | | Patient symptoms | | Mention NYHA class and specify if patient is asymptomatic or if patient has Dyspnea, PND, Orthopnea, Palpitation, Pedal edema, Chest pain, Syncope,Others |
| N 2 | | Clinical signs and ECG | | Mention height, weight, Respiratory rate, Heart rate, Blood pressure, SPO2, CVS, JVP, RS, ECG |
| N 3 | | Echocardiographic assessment | | Details of focused echocardiogram to be mentioned |
| N 4 | | TNPHDR Risk category | | Mention TNPHDR Risk category |
| N 5 | | Hospitalisations after discharge | | Mention any hospitalization after discharge |
| N 6 | | Post-partum plan | | Mention the postpartum treatment details and any interventions if suggested |
| N 7 | | **NEONATAL OUTCOME** | | Mention the neonatal weight gain, any echo evaluation done any adverse events |
| N 8 | | Date of next follow up: | | Mention the date when the patient is supposed to come for next visit |
| **O. FOLLOW UP VISIT -3** | | | | |
| O 1 | | Patient symptoms | | Mention NYHA class and specify if patient is asymptomatic or if patient has Dyspnea, PND, Orthopnea, Palpitation, Pedal edema, Chest pain, Syncope,Others |
| O 2 | | Clinical signs and ECG | | Mention height, weight, Respiratory rate, Heart rate, Blood pressure, SPO2, CVS, JVP, RS, ECG |
| O 3 | | Echocardiographic assessment | | Details of focused echocardiogram to be mentioned |
| O 4 | | TNPHDR Risk category | | Mention TNPHDR Risk category |
| O 5 | | Hospitalisations after discharge | | Mention any hospitalization after discharge |
| O 6 | | Post-partum plan | | Mention the postpartum treatment details and any interventions if suggested |
| O 7 | | **NEONATAL OUTCOME** | | Mention the neonatal weight gain, any echo evaluation done any adverse events |
| O 8 | | Date of next follow up: | | Mention the date when the patient is supposed to come for next visit |

| **TABLE II: MODIFIED WHO CLASSIFICATION** | | | | |
| --- | --- | --- | --- | --- |
| **mWHO I** | **mWHO II** | **mWHO II-III** | **mWHO III** | **mWHO IV** |
| - Small or mild   – pulmonary stenosis  – patent ductus arteriosus  – mitral valve prolapse   - Successfully repaired simple lesions (atrial or ventricular septal defect, patent ductus arteriosus, anomalous pulmonary venous drainage) - Atrial or ventricular ectopic beats, isolated | - Unoperated atrial or ventricular septal defect - Repaired tetralogy of Fallot - Most arrhythmias (supraventricular arrhythmias) - Turner syndrome without aortic dilatation | - Mild left ventricular impairment (EF >45%) - Hypertrophic cardiomyopathy - Native or tissue valve disease not considered WHO I or IV (mild mitral stenosis, moderate aortic stenosis) - Marfan or other HTAD syndrome without aortic dilatation - Aorta <45 mm in bicuspid aortic valve pathology - Repaired coarctation - Atrioventricular septal defect | - Moderate left ventricular impairment (EF 30–45%) - Previous peripartum cardiomyopathy without any residual left ventricular impairment - Mechanical valve - Systemic right ventricle with good or mildly decreased ventricular function - Fontan circulation. - If otherwise the patient is well and the cardiac condition uncomplicated - Unrepaired cyanotic heart disease - Other complex heart disease - Moderate mitral stenosis - Severe asymptomatic aortic stenosis - Moderate aortic dilatation (40–45 mm in Marfan syndrome or other HTAD; 45–50 mm in bicuspid aortic valve, Turner syndrome ASI 20–25 mm/m^2^, tetralogy of Fallot <50 mm) - Ventricular tachycardia | - Pulmonary arterial hypertension - Severe systemic ventricular dysfunction (EF <30% or NYHA class III–IV) - Previous peripartum cardiomyopathy with any residual left ventricular impairment - Severe mitral stenosis - Severe symptomatic aortic stenosis - Systemic right ventricle with moderate or severely decreased ventricular function - Severe aortic dilatation (>45 mm in Marfan syndrome or other HTAD, >50 mm in bicuspid aortic valve, Turner syndrome ASI >25 mm/m^2^, tetralogy of Fallot >50 mm) - Vascular Ehlers–Danlos - Severe (re)coarctation - Fontan with any complication |

**TABLE III: TESTED RISK SCORES**

| CARPREG I Score (19) | | CARPREG II Score (20) | | ZAHARA Score (21) | |
| --- | --- | --- | --- | --- | --- |
| Prior cardiac events before pregnancy (HF, Stroke, TIA, or Arrhythmia)  Baseline NYHA> 2 or cyanosis SPO2<90%  Left heart obstruction (Mitral Valve < 2 cm2, AVA < 1.5 cm2, Peak LV outflow tract pressure gradient P> 30 mm of Hg by echo  Reduced LV ejection fraction < 40% | -1  -1  -1  -1 | Prior cardiac events or arrhythmia  Baseline NYHA III-IV or cyanosis  Mechanical heart valve  Systemic LV dysfunction*****  High risk valve disease **^#^**  Pulmonary hypertension (RVSP > 49 mm of Hg)  High risk Aortopathy  Coronary Artery Disease  No prior Cardiac Intervention  Late pregnancy Assessment | -3  -3  -3  -2  -2  -2  -2  -2  -1  -1 | Mechanical heart valve  Left heart obstruction**^$^**  Prior Cardiac arrhythmia  Cardiac medication use before pregnancy  Cyanotic CHD  NYHA ≥ 2  Systemic AV Valve Regurgitation  Pulmonary AV Valve Regurgitation | -4.5  -2.5  -1.5  -1.5  -1  -0.75  -0.75  -0.75 |
| * LV ejection fraction <55%; **^#^** Aortic Valve area < 1.5 cm^2^ or subaortic gradient > 30 mm of Hg or Mitral stenosis < 2.0 cm^2^ or moderate to severe mitral regurgitation; **^$^** Pressure Gradient > 50 mm of Hg or AVA <1 cm^2^ | | | | | |
| HF-Heart Failure; TIA-Transient Ischemic Attack, AVA-Aortic valve area; LV- Left ventricle; RVSP-right ventricular systolic pressure; AV-Atrioventricular valve; CHD-Congenital Heart disease | | | | | |

| **TABLE IV: TNPHDR RISK SCORE** | | |
| --- | --- | --- |
| **TNPHDR I**  **(Low Risk)** | **THPHDR II**  **(Medium Risk)** | **TNPHDR III**  **(High Risk)** |
| Valvular heart disease   - Aortic stenosis- mild (aortic velocity<3.0 m/sec, mean aortic gradient <20 mm of Hg) - Mitral stenosis mild –valve area> 1.5 cm^2^ - Tricuspid stenosis mild- Mean gradient < 5 mm of Hg, Pressure half time < 190 milliseconds - Pulmonary stenosis - mild and moderate < 64 mm of Hg - Mild and moderate regurgitations of aortic, mitral, pulmonary & tricuspid valves | Valvular heart disease   - Aortic stenosis- moderate (aortic velocity 3.0-3.9 m/sec, mean aortic gradient 20-39 mm of Hg) - Aortic stenosis- severe asymptomatic (aortic velocity ≥4 m/sec or mean aortic gradient ≥ 40 mm of Hg) - Mitral stenosis : MVO > 1 to 1.5 cm^2^ - Tricuspid stenosis severe- Mean gradient ≥ 5 mm of Hg, Pressure half time ≥ 190 milliseconds - PS:Severe Peak gradient ≥ 64mm of hg - Severe regurgitation of aortic, mitral, pulmonary & tricuspid valves - Bio prosthetic Valve | Valvular heart disease   - Aortic stenosis- severe symptomatic. Aortic velocity ≥4 m/sec, mean aortic gradient ≥ 40 mm of Hg. - Mitral stenosis - severe (valve area ≤1.0) - Mechanical prosthetic Valve |
| - Left ventricular systolic dysfunction mild(LV ejection fraction > 45-54%) | - Left ventricular systolic dysfunction moderate(LV ejection fraction 45-31% %) | - Left ventricular systolic dysfunction severe (LV ejection fraction ≤30%) |
| - Previous PPCMP with no residual LV dysfunction in stress echo | - Previous PPCMP with no residual LV dysfunction (Stress echo not done) | - Previous PPCMP any residual LV dysfunction |
|  | - Hypertrophic cardiomyopathywith or without LVOT obstruction | - Right heart failure / Right ventricular dysfunction |
| - Isolated VPC and supra ventricular arrhythmias, SP PPI, Cong CAVB | - Ventricular tachycardia |  |
| - Mild PHT   TRPG >45 mmHg  RVSP >50 mmHg | - Moderate PHT   TRPG: 45-60 mmHg  RVSP : 50-65 mmHg | - Severe PAH   TRPG > 60 mmHg  RVSP > 65 mmHg |
| Pericardial effusion   - Mild or moderate without associated cardiac | Pericardial effusion   - Severe, Complicated. |  |
| - Connective tissue disease and vasculitis: graded by PA pressure and LV / RV function. |  | Vascular Ehlers Danlos |
|  | - Aorta<45 mm in Bicuspid A valve, - Aorta < 40mm in Marfan’s | - Aorta ≥ 45 mm in Bicuspid A valve, - Aorta ≥ 40mm in Marfan’s |
|  | - Repaired Co A - Native coarctation < 20 mg gradient | - Re-coarctation - Severe Native Coarctation |
| - Native shunts without risk modifiers - Operated shunts | - Native shunts with risk modifier’s |  |
| - Repaired TOF and TOF physiology - Asymptomatic Ebstein’s | - Unrepaired TOF / TOF physiology - Repaired complex CCHD - Normally functioning Systemic RV - Uncomplicated Fontan - Symptomatic Ebstein’s | - Unrepaired complex CCHD - Systemic RV with dysfunction - Complicated Fontan |
| - Isolated dextrocardia |  |  |
| Note: If a given patient has multiple risk determining conditions, the patient is assigned to the highest risk group condition | | |

| **TABLE V. DRUG PAGE-USE AND DOSAGE** | | | | | | | | | | | | | | |
| --- | --- | --- | --- | --- | --- | --- | --- | --- | --- | --- | --- | --- | --- | --- |
| **NO** | **DRUG** | **E1**  **PRE- PREGNANCY** | | **E2**  **I TRIMESTER** | | | **E3**  **II TRIMESTER** | | | **E4**  **III TRIMESTER** | | | **E5**  **PERIPARTUM** | |
|  |  |  | **DOSAGE** |  | **DOSAGE** |  | | **DOSAGE** |  | | **DOSAGE** |  | | **DOSAGE** |
| 1 | Penicillin |  |  |  |  |  | |  |  | |  |  | |  |
| 2 | Warfarin |  |  |  |  |  | |  |  | |  |  | |  |
| 3 | Acenocoumarol |  |  |  |  |  | |  |  | |  |  | |  |
| 4 | Phenindione |  |  |  |  |  | |  |  | |  |  | |  |
| 5 | Aspirin |  |  |  |  |  | |  |  | |  |  | |  |
| 6 | Clopidogrel |  |  |  |  |  | |  |  | |  |  | |  |
| 7 | Nitrates |  |  |  |  |  | |  |  | |  |  | |  |
| 8 | Atorvastatin |  |  |  |  |  | |  |  | |  |  | |  |
| 9 | Rosuvastatin |  |  |  |  |  | |  |  | |  |  | |  |
| 10 | Verapamil |  |  |  |  |  | |  |  | |  |  | |  |
| 11 | Nifedipine |  |  |  |  |  | |  |  | |  |  | |  |
| 12 | Diltiazem |  |  |  |  |  | |  |  | |  |  | |  |
| 13 | Atenolol |  |  |  |  |  | |  |  | |  |  | |  |
| 14 | Metoprolol |  |  |  |  |  | |  |  | |  |  | |  |
| 15 | Other BBs |  |  |  |  |  | |  |  | |  |  | |  |
| 16 | Digoxin |  |  |  |  |  | |  |  | |  |  | |  |
| 17 | Frusemide |  |  |  |  |  | |  |  | |  |  | |  |
| 18 | Spironolactone |  |  |  |  |  | |  |  | |  |  | |  |
| 19 | Hydrochlorothiazide |  |  |  |  |  | |  |  | |  |  | |  |
| 20 | Enalapril |  |  |  |  |  | |  |  | |  |  | |  |
| 21 | Ramipril |  |  |  |  |  | |  |  | |  |  | |  |
| 22 | Other ACEIs |  |  |  |  |  | |  |  | |  |  | |  |
| 23 | Losartan |  |  |  |  |  | |  |  | |  |  | |  |
| 24 | Telmisartan |  |  |  |  |  | |  |  | |  |  | |  |
| 25 | Other ARBs |  |  |  |  |  | |  |  | |  |  | |  |
| 26 | Sildenafil |  |  |  |  |  | |  |  | |  |  | |  |
| 27 | Bosentan |  |  |  |  |  | |  |  | |  |  | |  |
| 28 | Amiodarone |  |  |  |  |  | |  |  | |  |  | |  |
| 29 | Oral anti-diabetics |  |  |  |  |  | |  |  | |  |  | |  |
| 30 | Insulin |  |  |  |  |  | |  |  | |  |  | |  |
| 31 | Other drugs |  |  |  |  |  | |  |  | |  |  | |  |

| **TABLE VI. ANTICOAGULANT/ANTIPLATELET REGIMEN** | | | | | | | | | | | | | | | | | | | |
| --- | --- | --- | --- | --- | --- | --- | --- | --- | --- | --- | --- | --- | --- | --- | --- | --- | --- | --- | --- |
| Pre-pregnancy | ACITROM WARFARIN Average INR_____ ASPIRIN | | | | | | | | | | | | | | | | | | |
| Present pregnancy | 1st trimester | | | 2nd trimester | | | 3rd trimester (upto 36wks) | | | Peripartum (>36 wks) | | | Intrapartum | | | Postpartum | | Follow up | |
| Oral anti-  Coagulant | Dose | INR | Period | Dose | INR | Period | Dose | INR | Period | Dose | INR | Period | Dose | INR | Period | Dose | INR | Period | Dose |
| Acitrom |  |  |  |  |  |  |  |  |  |  |  |  |  |  |  |  |  |  |  |
| Warfarin |  |  |  |  |  |  |  |  |  |  |  |  |  |  |  |  |  |  |  |
| Parental anti-coagulant | Dose | APTT | Period | Dose | APTT | Period | Dose | APTT | Period | Dose | APTT | Period | Dose | APTT | Period | Dose | APTT | Period | Dose |
| UFH | Infusion  Subcut  IV Bolus | | | Infusion  Subcut  IV Bolus | | | Infusion  Subcut  IV Bolus | | | Infusion  Subcut  IV Bolus | | | Infusion  Subcut  IV Bolus | | | Infusion  Subcut  IV Bolus | | Infusion  Subcut  IV Bolus | |
| APTT/ANTI XA |  | | |  | | |  | | |  | | |  | | |  | |  | |
| Duration |  | | |  | | |  | | |  | | |  | | |  | |  | |
|  | Dose | APTT | Period | Dose | APTT | Period | Dose | APTT | Period | Dose | APTT | Period | Dose | APTT | Period | Dose | APTT | Period | Dose |
| LMWH  ENOXAPARIN  DALTEPARIN |  |  |  |  |  |  |  |  |  |  |  |  |  |  |  |  |  |  |  |
| ASPIRIN | No Yes, Dose____ | | | No Yes, Dose____ | | | No Yes, Dose____ | | | No Yes, Dose____ | | | No Yes, Dose____ | | | No Yes Dose____ | | No Yes Dose____ | |
